# EFFECTIVENESS OF PHYSICAL, PSYCHOLOGICAL, AND ALTERNATIVE INTERVENTIONS FOR POST-COVID-19 CONDITION: A SYSTEMATIC REVIEW

**DOI:** 10.2340/jrm.v58.46279

**Published:** 2026-10-19

**Authors:** Akram NEGM, Maike STOLZ, Christian KRAUTH

**Affiliations:** Institute of Epidemiology, Social Medicine and Health System Research, Hannover Medical School, Hannover, Germany

**Keywords:** exercise therapy, fatigue, post-acute COVID-19 syndrome, quality of life, rehabilitation

## Abstract

**Objective:**

To evaluate supervised physical, psycho-logical, and alternative non-pharmacological interventions for improving patient-reported and objective functional outcomes in adults with post-COVID-19 condition.

**Design:**

Systematic review of randomized controlled trials.

**Methods:**

PubMed, Web of Science Core Collection, APA PsycInfo, and Scopus were searched from 1 January 2020 to 29 January 2026. Eligible studies were supervised, face-to-face trials evaluating physical, psychological, or alternative non-pharmacological interventions against usual care, placebo/sham, or active comparators in adults with World Health Organization defined post-COVID-19 condition. Risk of bias was assessed using Cochrane RoB 2. Due to clinical and methodological heterogeneity, a narrative synthesis was performed.

**Results:**

27 trials (1,998 participants) were included and evaluated physical, psychological, and alternative therapies. Supervised cardiopulmonary rehabilitation and multimodal exercise consistently improved cardiorespiratory fitness and walking capacity. Cognitive-behavioural therapy and lifestyle adjustments significantly reduced fatigue and subjective concentration complaints. Findings for neuromodulation, complementary, and oxygen therapies were heterogeneous, often showing no benefit over placebo. Only 18.5% of trials had low risk of bias, limited by small samples, unblinding, and short follow-ups.

**Conclusion:**

Supervised physical rehabilitation and CBT show promising evidence for improving functional capacity and symptoms in post-COVID-19 condition. Larger trials with longer follow-up are needed to confirm effectiveness and inform clinical guidelines.

## LAY ABSTRACT

Many people continue to feel unwell for months after a COVID-19 infection. This is known as post-COVID-19 condition. Common problems include tiredness, shortness of breath, reduced fitness, problems with concentration, and difficulty returning to normal daily life. We reviewed 27 randomized controlled trials to find out which non-pharmacological treatments may help. These included exercise programmes, breathing exercises, lifestyle coaching, psychological support, and newer methods like oxygen therapy. Overall, active and structured rehabilitation worked best. Programs that combined fitness training, strength exercises, and breathing work often helped people walk further, breathe easier, feel less fatigued, and enjoy a better quality of life. Breathing exercises and cognitive-behavioural therapy were also helpful for specific symptoms. Our findings show that post-COVID-19 condition can improve with the right support. Doctors and therapists should offer tailored rehabilitation to help patients return to their daily lives.

Post-COVID-19 condition (PCC), is defined by the World Health Organization as the continuation or development of new symptoms 3 months after an initial SARS-CoV-2 infection, persisting for at least 2 months without an alternative explanation (1). PCC has emerged as a major global health concern, affecting an estimated 400 million people worldwide, with prevalence rates of approximately 6–7% in adults and 1% in children (2). The risk of developing PCC is higher in women, older adults, smokers, individuals with obesity or pre-existing chronic diseases, and those experiencing repeated infections or severe acute illness requiring hospitalization or intensive care. Socioeconomic determinants of health and insufficient rest during the early phase of COVID-19 may also contribute (3). PCC manifests with a wide range of symptoms; over 200 have been reported, including fatigue, musculoskeletal pain, dyspnoea, headaches, cognitive impairment, altered taste, impaired sleep, depression, and anxiety (4). PCC significantly impairs quality of life and can limit daily functioning and the ability to work, highlighting its broader social and economic consequences (5, 6).

Recent evidence suggests that non-pharmacological interventions for PCC offer promising, though uneven, benefits across outcomes and delivery models. Early systematic reviews, using GRADE methodology, rated the evidence for pulmonary rehabilitation and self-directed activities as very low certainty, primarily due to high risk of bias, substantial heterogeneity across interventions and outcomes, and imprecision related to small sample sizes (7). More recent randomized trials indicate moderate certainty improvements in functional exercise capacity and quality of life, with lower certainty for dyspnoea, fatigue, and respiratory function (8). Pulmonary rehabilitation that combines aerobic and resistance training with breathing components appears to benefit dyspnoea, quality of life, and mood, while effects on fatigue remain mixed. Targeted respiratory muscle training also improves pulmonary measures, dyspnoea, and functional capacity (9, 10). Emerging options such as non-invasive brain stimulation and alternative or complementary therapies show preliminary signals but are limited by small samples and low evidence certainty (11, 12).

Evidence from systematic reviews that comprehensively evaluate all non-pharmacological interventions in patients with post-COVID-19 condition remains limited. The aim of this systematic review was to examine the effectiveness of physical, psychological, and alternative interventions in improving patient-relevant and functional outcomes among adults with post-COVID-19 condition.

## METHODS

### Review protocol

This systematic review was conducted in accordance with the Preferred Reporting Items for Systematic Reviews and Meta-Analyses (PRISMA) 2020 (13). The review protocol was prospectively registered in the International Prospective Register of Systematic Reviews (PROSPERO) under the registration number CRD420251088492.

### Eligibility criteria

The criteria for inclusion were: *(i)* adults with post-COVID-19 condition (PCC) as defined by the World Health Organization; *(ii)* randomized controlled trials; *(iii)* physical, psychological, or alternative non-pharmacological intervention trials; *(iv)* interventions delivered in a supervised, face-to-face clinical or rehabilitation setting; *(v)* comparison with usual care, placebo, sham, or an alternative non-pharmacological intervention; and *(vi)* reporting on at least 1 patient-reported outcome.

To reflect the comparator heterogeneity throughout the manuscript, we distinguish 3 control-condition categories: trials comparing an eligible intervention with “usual care”; trials comparing an eligible intervention with “placebo/sham”, and trials comparing an eligible intervention with an “active comparator”. These terms are used consistently throughout the Results and Discussion to specify the control condition against which each between-group finding was obtained.

Studies were excluded if they: *(i)* did not meet the WHO definition for PCC; *(ii)* investigated pharmacological interventions only; *(iii)* used non-randomized study designs; or *(iv)* evaluated interventions delivered virtually, home-based, or in other unsupervised formats.

Given the clinical and methodological heterogeneity across non-pharmacological interventions in post-COVID-19 condition, eligibility criteria were refined to include only supervised, face-to-face interventions. Remote or unsupervised interventions were excluded because differences in monitoring, adherence, exercise prescription control, and real-time adjustment may influence treatment effects and introduce additional heterogeneity.

### Information sources and search strategy

A systematic literature search was conducted in accordance with the PICO framework. The databases PubMed, Web of Science Core Collection, APA PsycInfo, and Scopus were searched for records published between 1 January 2020 and 29 January 2026. Search strategies combined controlled vocabulary (e.g., MeSH terms) and free-text synonyms for post-COVID-19 condition, non-pharmacological interventions, and relevant outcomes. The strategy was designed and run by the first reviewer (AN). No restrictions were applied regarding publication type or language. Additional searches included manual screening of reference lists of all eligible studies. All sources were last consulted on 29 January 2026. The full PubMed search strategy is provided in Table SI and was adapted for use in the other databases.

### Selection process

Prior to screening, duplicate records were removed. Titles and abstracts were then independently screened by 2 reviewers (AN, MS) using Rayyan (14). Full texts of potentially eligible reports were subsequently retrieved and assessed for inclusion by the same reviewers. Discrepancies at either stage were resolved through discussion, and, when necessary, adjudicated by a third reviewer (CK). Final inclusion required consensus between the 2 primary reviewers (AN, MS). The study selection process is illustrated in the PRISMA 2020 Flowchart (Fig. 1), and a list of excluded full-text articles with reasons is provided in Table SII.

**Fig. 1:**
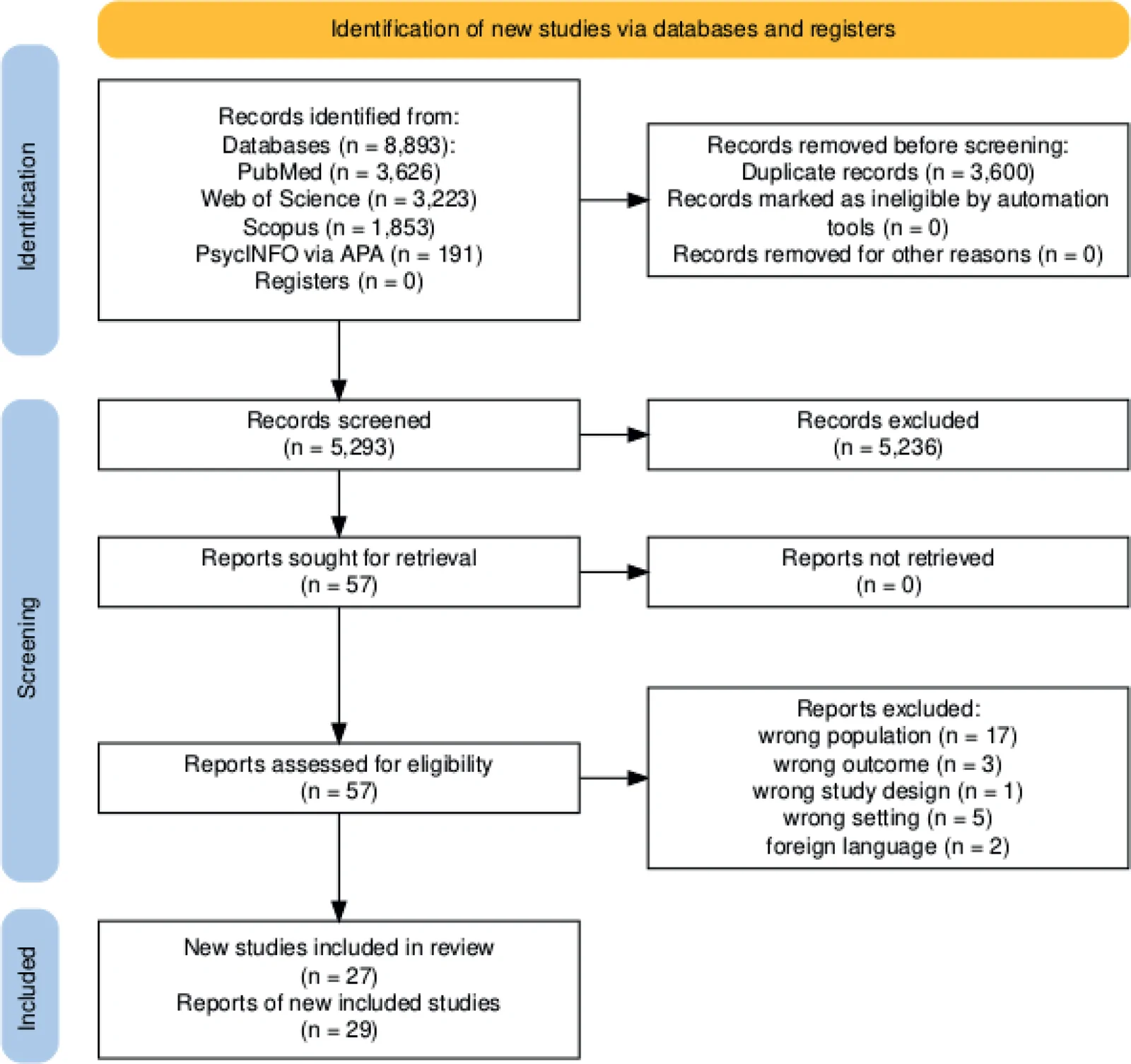
PRISMA 2020 flowchart.

### Data collection process

Data from each included report were extracted by 1 reviewer (AN) using a standardized form piloted on 3 studies, and all extracted data and findings were documented in a standardized Excel workbook (S3) (Microsoft Corp, Redmond, WA, USA). A second reviewer (MS) independently checked key fields and values for accuracy. Reviewers worked independently and resolved discrepancies by discussion, with a third reviewer (CK) available if needed.

### Data items

From each included study, we extracted information on authors, year of publication, country, study setting, participant characteristics, intervention details, and comparator characteristics. Outcomes were categorized into predefined domains. Within each domain, all relevant outcomes reported by a study were collected. Where necessary, standard conversions were applied to harmonize data for synthesis.

### Study risk of bias assessment

Risk of bias was assessed for each included RCT using the Cochrane Risk of Bias 2 (RoB 2) tool (15). Two reviewers (AN, MS) independently evaluated the 5 RoB 2 domains: bias arising from the randomization process, deviations from intended interventions, missing outcome data, measurement of the outcome, and selection of the reported result following the tool’s signalling questions and decision rules. Domain-level judgements were categorized as low risk, some concerns, or high risk, and an overall study-level judgement was derived accordingly. Disagreements were resolved by discussion, with a third reviewer available if needed.

### Synthesis methods

We conducted a qualitative narrative analysis to describe the effectiveness of physical, psychological, and alternative interventions in improving patient-relevant and functional outcomes among adults with PCC. For synthesis, included studies were categorized by type of intervention. Given the methodological, clinical, and statistical heterogeneity, it was not possible to conduct a meta-analysis.

## RESULTS

### Study selection

The database search identified 8,893 records from 4 databases. After removing 3,600 duplicates, 5,293 records remained for title and abstract screening, of which 5,236 were excluded. We sought and assessed 57 full-text reports for eligibility. Of these, 28 were excluded at full text. Finally, 27 studies met the inclusion criteria and were included in the review, corresponding to 29 reports. Studies that initially appeared eligible but were excluded enrolled populations outside WHO-defined PCC. The list of excluded studies is reported in Table SII and the overall process is summarized in Fig. 1 (16). For study characteristics, see Table I.

**Table I:** Characteristics of included studies

Author and year	Population	Intervention	Control	Outcome	Main results
Ahmad M M et al., 2024	Adults with PCC *n* = 16 IG: *n* = 8 (80% F) Mean age: 49.38 ± 12.24 y/o CG: *n* = 8 (90% F) Mean age: 50.0 ± 11.06 y/o	Continuous treadmill walking/jogging. VR group: non-immersive VR scene (Wii Fit Plus) displayed in front of treadmill with motion interaction; supervised by a physiotherapist Duration: 4 weeks, 3 sessions/week; each session 30–45 min active phase Intensity: Moderate intensity set by Karvonen: THR = 50–60% (HR_peak − HR_rest) + HR_rest; individualized HR monitoring; volume progressed so all participants trained at the upper THR by end of programme	Active comparator: identical treadmill protocol without VR (conventional aerobic exercise); supervised by a physiotherapist	Primary: Fatigue (CFS) Cognitive function (MoCA) Sleep quality (PSQI) Secondary: Satisfaction (participant satisfaction with exercise programme)	Both groups showed significant within-group improvements in fatigue, cognitive function, and sleep quality after 4 weeks (all *p* < 0.05). However, there were no significant between-group differences between VR-assisted and conventional treadmill exercise for fatigue (*p* = 0.063), cognitive function (*p* = 0.640), or sleep quality (*p* = 0.921)
Bai et al., 2024	Adults with PCC *n* = 24 IG = 12 (33.3% F) Mean age: 46.85 ± 15.26 y/o CG = 12 (83.3% F) Mean age: 43.42 ± 14.96 y/o	Supervised, individualized aerobic exercise training on cycling ergometers (MIIT and HIIT protocols) Duration: 4 weeks; 3 supervised sessions/week MIIT: 4 cycles of 8-min exercise/2-min rest at 40%→55% peak WR. HIIT: 30-s/30-s intervals at 80%→90% peak WR Intensity: individualized via baseline CPET-derived VO_2_peak and peak WR. RPE, HR, BP, and SpO_2_ monitored continuously throughout sessions. Sessions included a 5-min warm-up and 5-min cool-down at 20% peak WR	Usual care: guideline-based healthy lifestyle recommendations and WHO self-management guidance after COVID-19-related illness	Maximal exercise capacity: VO_2_peak (mL·kg^–^¹·min^–^¹), maximum workload (W), exercise time Ventilatory efficiency: VE/VCO_2_ slope Submaximal threshold: VO_2_ at anaerobic threshold Symptom burden: Total long COVID-19 symptom count QoL: SF-12 (PCS, MCS) Psychological: PHQ-9, GAD-7, PSS Sleep: ISI Pulmonary function: FEV_1_, FVC, FEV_1_/FVC	Aerobic exercise training produced significantly greater improvements in cardiorespiratory fitness compared with usual care. VO_2_peak increased by +2.1 mL·kg^–^¹·min^–^¹ in the IG vs +0.1 mL·kg^–^¹·min^–^¹ in the CG, with a significant between-group difference (*p* < 0.001). Maximum workload (WR) improved significantly in favour of the IG (between-group Δ: +10.92 W) Long COVID symptom reduction was observed in 66.7% of IG participants vs 0% in the CG. Between-group differences significantly favoured the IG for anxiety (GAD-7: −3.58), depression (PHQ-9: −3.58), perceived stress (PSS: −4.75), SF-12 PCS (+6.55), and SF-12 MCS (+7.50) Pulmonary function parameters (FEV_1_, FVC) did not change significantly in either group
Barz et al., 2024	Adults with PCC *n* = 118 IG: *n* = 58 (65.5% F) Mean age IG: 52.8 ± 11.6 y/o CG: *n* = 60 (71.7% F) Mean age CG: 53.5 ± 12.3 y/o	Supervised, individualized symptom-titrated concurrent resistance + aerobic training Duration: 10 weeks (2-week familiarization + 8-week intervention); 1–3 sessions/week Resistance training: machine-based whole-body programme (8 exercises); 2 × 12 reps; cluster sets during higher fatigue Endurance training: ergometer-based aerobic exercise (5–30 min) Intensity: adjusted daily using Brief Fatigue Inventory (BFI); OMNI/RPE-based autoregulation	Usual care	Primary: Fatigue severity (FSS) Secondary: Health-related quality of life (SF-12 PCS, MCS) Post-exertional malaise (DSQ-PEM) Hand grip strength (Fmax, Fmean; fatigue & recovery ratios) Endurance capacity (Chester Step Test)	Symptom-titrated training resulted in a significantly greater reduction in fatigue compared with the control group. FSS decreased by −1.14 points in IG vs −0.23 in CG (*p* < 0.001), reaching clinical relevance
Bobowik et al., 2024	Older adults with PCC *n* = 46 IG = 26 Mean age: 69.39 ± 4.99 y/o CG = 20 Mean age: 74.25 ± 7.31 y/o	Supervised, progressive machine-based whole-body resistance training Duration: 8 weeks; 2 sessions/week; 60 min/session Exercises: incline bench press, 45° leg press, latissimus pull-down, trunk crunch, T-bar row, leg extension, leg curl; 3 sets × 12 reps at 70% 1RM (Brzycki formula) Load increased by 5 kg upon completion of all reps Warm-up: 15-min aerobic activity (treadmill/Orbitrek at 60–65% HRmax) Pre-session monitoring of HR, BP, and SpO_2_	Usual care	Primary: Postural stability – Biodex Balance System: Overall Stability Index (OSI), Anterior–Posterior Stability Index (APSI), Medial–Lateral Stability Index (MLSI), Fall-Risk Index (FRI 12–8; FRI 6–2) Secondary: Health-related QoL: WHOQOL-BREF (DOM1–4, Q1–Q2)	Resistance training significantly improved postural stability. OSI (eyes open) improved significantly in the IG with a large effect size (Z = −3.12, *p* < 0.01, R = 0.533); FRI 6–2 also improved significantly (Z = −2.06, *p* < 0.05, R = 0.354), indicating reduced fall risk
Besnier F. et al., 2024	Adults with PCC *n* = 35 IG: *n* = 18 (65%F) Mean age: IG: 52.54 ± 11.29 y/o CG: *n* = 17 (72%F) Mean age: 53.89 ± 12.13 y/o	Centre-based, individualized cardiopulmonary rehabilitation combining cycling-based aerobic training, machine-based resistance training and daily inspiratory muscle training Duration: 8 weeks; 3 supervised sessions/week plus daily respiratory exercises Intensity: RPE-guided 10–13; aerobic 10→20→30 min (incl. 5-min warm-up/cool-down); resistance 3×10 at OMNI-RES ~3–6; light daily IMT; HR/SpO_2_ monitored; adjust for post-exertional symptoms	Usual care	Primary: Maximal exercise capacity (VO_2_peak, mL·kg^–^¹·min^–^¹) Secondary: Ventilatory efficiency (VE/VCO_2_ slope) Submaximal thresholds (VO_2_ at VT_1_; VO_2_ at VT_2_, mL·kg^–^¹·min^–^¹) Oxygen uptake efficiency (OUES) Physical function (6MWT; TUG; 5STS) Quality of life (SF-36) Dyspnoea (MRC) Functional status (PCFS) Symptom impact (Long-COVID Symptom & Impact Tools)	Cardiopulmonary rehabilitation resulted in a significantly greater improvement in cardiorespiratory fitness compared with usual care VO_2_peak increased by +2.73 mL·kg^–^¹·min^–^¹ (95% CI +1.64 to +3.82; *p* < 0.001) in the rehabilitation group vs +0.28 mL·kg^–^¹·min^–^¹ (95% CI −0.78 to +1.33; *p* = 0.596) in controls; the between-group difference was significant (*p* = 0.003)
Cherkasova et al., 2025	Adults with post-COVID syndrome with neurological manifestations *n* = 20 IG = 10 (60% F) Mean age: 41 ± 13.33 y/o CG = 10 (90% F) Mean age: 31 ± 11.85 y/o	EEG α-rhythm biofeedback training using the NeuroPlay-6C neurofeedback system Duration: 21–27 days; 12–15 sessions on weekdays; 30–50 min/session Techniques: breath control, progressive muscle relaxation, mental training, and meditation (closed-eyes sessions with auditory feedback) Intensity: governed by real-time EEG α-rhythm monitoring: auditory noise increased when target α-activity was not achieved and ceased when desired rhythm was detected. Adaptive self-regulation training based on spectral EEG characteristics	Sham EEG biofeedback training (placebo neurofeedback)	Emotional state: STAI, SHAI, BDI, PSM-25 Cognitive function: ACE-III, Schulte tables, Stroop test, Tower of London, N-back test, 10-word memory test Fatigue (asthenia): MFI-20 (total and subscales) Sleep quality: ISI	Biofeedback significantly improved immediate verbal recall (10-word memory test; *p* = 0.036) and working memory (N-back, *n* = 3; *p* = 0.034) over sham. General fatigue reduction was significantly greater in the IG than the CG (MFI-20 general fatigue; *p* = 0.019), indicating superior efficacy for asthenia Both groups showed comparable improvements in emotional outcomes (anxiety, depression, psychological stress), with no significant between-group differences. Sleep quality (ISI) did not improve significantly in either group
D‘hoore et al., 2025	Adults with PCC *n* = 101 Group A = 29 (66% F) Group B = 29 (49% F) Group C = 22 (42% F) Group D = 21 (39% F) Mean ages: Group A: 43.7 ± 11.2 y/o Group B: 49.4 ± 10.3 y/o Group C: 50.0 ± 11.8 y/o Group D: 46.8 ± 8.2 y/o	Oxygen therapy delivered in pressure chambers at varying concentrations and pressures Duration:10 sessions over 2 consecutive weeks; 95 min/session (15-min compression, 70-min treatment, 10-min decompression) Group A: 100% O_2_ at 2.5 ATA (hyperbaric hyperoxia) Group B: 40% O_2_ at 2.5 ATA (hyperbaric hyperoxia with lower O_2_ concentration) Group C: 100% O_2_ at 1 ATA (normobaric hyperoxia)	Group D (placebo): 21% O_2_ (ambient air) at 1 ATA; identical session duration and procedure.	Primary: VAS symptom severity EQ-5D-5L modified COVID-19 Yorkshire Rehabilitation Scale (C19-YRSm) Secondary: Functional exercise capacity: 6MWT (distance, SpO_2_, Borg RPE) Neurocognitive battery: Digit Span Backward (DSB), Simple Reaction Time (REA), Math Processing Test (MathProc), Trail-Making Test (Ptrails), TimeWall Test	No significant differences were observed between any treatment group and placebo in subjective symptom severity (VAS), quality of life (EQ-5D-5L), or COVID-19 symptom burden (C19-YRSm) Functional exercise capacity (6MWT) and all neurocognitive parameters did not differ significantly between groups Comparable subjective improvement was reported across all arms including placebo, suggesting a predominant placebo effect or natural symptom fluctuation. At 3-month follow-up, most participants had returned to their baseline condition. Oxygen therapy at varying doses and pressures demonstrated no superior clinical benefit over placebo
Cabello Fernandez et al., 2025	Adults with PCC and persistent cognitive complaints *n* = 122 Cognitive IG = 63 (68.3% F) Mean age: 47.3 ± 9.8 y/o Affective CG = 59 (71.2% F) Mean age: 46.7 ± 10.5 y/o	Cognitive-focused psychoeducational programme targeting metacognition and compensatory cognitive strategies Duration: 4 weekly sessions of 90 min + 30-min reactivation session at 1 month post-intervention Modules: fatigue and sleep management, working memory and attention strategies, executive function (planning, goal management), and long-term memory strategies Techniques: sleep hygiene, fatigue pacing, environmental adjustments, time pressure management, goal management training, internal/external memory aids Sessions supported by video material and home exercises	Active comparator: affective psychoeducational programme focused on emotional regulation, coping strategies, and behavioural activation	Primary: Cognitive complaints (BRIEF-A: GEC; MMQ composite) – T1 (2 months) Secondary: Cognitive complaints (BRIEF-A: GEC, BRI, MI; MMQ composite, Satisfaction, Ability, Strategies) – T2 (8 months) Objective cognitive performance (attention, working memory, long-term memory, executive functions, language) Fatigue (MFIS-21: total, physical, cognitive, psychosocial) Sleep quality (PSQI) Psychological distress (OQ-45: total, symptom distress, interpersonal relations, social role) Quality of life (QLSI; EQ-5D-5L VAS) Work and activity impairment (WPAI: work impairment, activity impairment)	Both groups showed significant reductions in subjective cognitive complaints at 2 months. BRIEF-A (GEC) scores improved significantly over time (F = 17.4, *p* < 0.001), with small-to-moderate effect sizes in both arms (cognitive: *d* = 0.48, *p* = 0.008; affective: *d* = 0.59, *p* = 0.002). MMQ composite scores similarly improved (F = 16.33, *p* < 0.001; cognitive: *d* = 0.49, *p* = 0.007; affective: *d* = 0.54, *p* = 0.004) No group-by-time interaction was found for either measure, indicating that the two interventions did not differ significantly in their effects on the primary outcome. Reliable change indices showed that approximately one-third of patients in each group improved beyond measurement error (BRIEF-A: 37% cognitive, 39% affective; MMQ: 35% cognitive, 36% affective)
Gomes dos Santos E.G. et al., 2024	Adults with PCC *n* = 33 IG: *n* = 17 (58.82% F) Mean age: 50.76 ± 11.28 y/o CG: *n* = 16 (62.5 %) Mean age: 44.0 ± 11.28 y/o	Cardiopulmonary rehabilitation combining respiratory therapy (EPAP/PEEP + IMT), resistance (upper-limb), and aerobic treadmill training Duration: 6 weeks Intensity: Lung expansion: EPAP/PEEP 5–20 cmH_2_O, 3×2 min (↑5 cmH_2_O if Borg 4–6). IMT (Threshold®): 3×10 reps, 40%→50% MIP (weeks 1–3 → 4–6). Upper-limb strengthening: 3×1 min shoulder flexion; load ↑ 0.5 kg if Borg 4–6. Aerobic: treadmill 20 min at 60–70% HR (weeks 1–3) → 70–80% HR (weeks 4–6). HR/SpO_2_ monitored; O_2_ given if SpO_2_ ≤ 90%	Usual care/education: bi-weekly remote lectures for 6 weeks with symptom follow-up; encouraged daily activities	Primary: Physical function (6MWT) Secondary: Dyspnoea (mMRC) Fatigue (MFIS) Muscle mass (kg; upper and lower limbs)	After 6 weeks, cardiopulmonary rehabilitation resulted in a significantly greater improve-ment in functional capacity compared with the control group. The distance walked on the 6MWT increased by +199.29 m (95% CI 99.45 to 299 m) in the rehabilitation group vs +61.62 m (95% CI −41 to 164 m) in controls; the between-group difference was +100.46 m (95% CI 7.40 to 193 m; *p* = 0.002)
Jimeno-Almazán et al., 2023	Non-hospitalized adults with PCC *n* = 80 CT group: *n* = 20 (70% F) Mean age: 44.3 ± 9.6 y/o RM group: *n* = 17 (59% F) Mean age: 43.0 ± 6.9 y/o CT + RM group: *n* = 23 (74% F) Mean age: 44.1 ± 7.4 y/o CG: *n* = 20 (70% F) Mean age: 47.8 ± 7.6 y/o	Concurrent training (CT): supervised multicomponent aerobic + resistance CT + RM: CT plus inspiratory muscle training RM: stand-alone inspiratory muscle training Duration: 8 weeks CT/CT + RM: 3 supervised sessions/week (2 concurrent days + 1 autonomous LICT day) RM: 2 sessions/day, 7 days/week. CG: home programme throughout 8 weeks Intensity: Resistance (CT/CT + RM): 50% 1RM, 3×8 reps, 4 exercises (squat, bench press, deadlift, bench pull) Endurance (CT/CT + RM): MIVT 4–6×(3–5 min at 70–80% HRR / 2–3 min at 55–65% HRR); LICT 30–60 min at 65–70% HRR (RPE targets: 11–12 for LICT/strength; ≤16 for MIVT) RM (IMT): PowerBreath® 1×30 reps, twice daily at ~62.5% PIM, load ↑ every 2 wks to keep RPE 12–15. HR monitored; velocity-based load control for strength Attendance threshold ≥ 85%	WHO self-management leaflet (“Support for Rehabilitation: Self-Management after COVID-19-Related Illness”); unsupervised, adherence not monitored	Primary: Cardiorespiratory fitness (VO_2_max, mL/kg/min) Muscle strength (upper and lower body strength; handgrip strength) Secondary: Quality of life (SF-12) Anxiety (GAD-7) Depression (PHQ-9) Dyspnoea (mMRC) Fatigue (FSS; CFS) Functional status (PCFS scale)	After 8 weeks, there were no significant between-group differences in estimated V˙O_2_max; however, significant within-group improvements were observed in CT (+7.5%) and CT+RMT (+7.8%) Lower-body strength improved significantly in the concurrent training arms compared with inspiratory muscle training alone and control (CT/CT+RMT: +14.5% to +32.6% vs RM/CON: −0.3% to +11.3%, *p* < 0.05 for group effects)
Kaddoussi et al., 2024	Adults with PCC and persistent dyspnoea (mMRC ≥ 2) *n* = 30 IG = 20 (50% F) Mean age: 53 ± 14 y/o CG = 10 (60% F) Mean age: 52 ± 14 y/o	Ambulatory cardiopulmonary rehabilitation programme (CPRP) Duration: 6 weeks; 3 sessions/week; 18 sessions; 60–90 min/session Components per session: warm-up (~5 min), progressive aerobic treadmill training (10→35 min), resistance training – 5 exercises (leg extension, heel raises, sit–stand, overhead press, biceps curls; 15–20 min), and respiratory exercises (controlled breathing, chest expansion; ~15 min). Therapeutic education also provided. Intensity: individualized using HR at end of 6MWT ± 5 bpm as target training HR; continuously monitored throughout sessions	Usual care	Primary: Submaximal aerobic capacity (6MWD) Secondary: Dyspnoea: mMRC scale, Borg scale Spirometry: FEV_1_, FVC, FEV_1_/FVC ratio Physiological: resting HR, SpO_2_, 6-minute walk work (6MWW)	Cardiopulmonary rehabilitation produced a clinically and statistically significant improvement in submaximal exercise capacity. 6MWD increased by +168 ± 99 m (~28%) in the IG vs +5 ± 45 m in the CG, exceeding the MCID of 30 m
Kjellberg et al., 2025	Adults with PCC *n* = 80 HBOT = 40 (80% F) Mean age: 41.1 ± 10.1 y/o Sham = 40 (82.5% F) Mean age: 41.4 ± 8.2 y/o	Hyperbaric oxygen therapy (HBOT): 100% medical O_2_ via mask at 2.4 ATA Duration: 10 sessions over 6 weeks (every 2–3 days); 90 min/session with two 5-min air breaks Administered in monoplace hyperbaric chambers	Sham hyperbaric treatment: medical air (instead of O_2_); initial brief pressurization to 1.34 ATA then maintained at 1.2 ATA; identical 90-min session duration with 2 x 5-min air breaks	Primary: RAND-36 Physical Functioning (PF) and Role Limitations due to Physical Health (RP) at 13 weeks Secondary: Functional exercise capacity: 6MWT Lower-limb strength: 30-second Chair–Stand Test (CST) Health-related QoL: EQ-5D-5L Endothelial function: Reactive Hyperaemia Index (RHI)	HBOT did not produce significantly greater improvements than sham on any primary or secondary outcome RAND-36 PF: between-group difference 0.63 points (95% CI −7.04 to 8.29; *p* = 0.87). RAND-36 RP: difference 2.35 points (95% CI −5.95 to 10.66; *p* = 0.57)
Kroll et al., 2025	Adults with PCC undergoing inpatient pulmonary rehabilitation *n* = 40 IG = 20 Mean age: 50 ± 13 y/o CG = 20 Mean age: 52 ± 11 y/o	Cranial Electrotherapy Stimulation (CES) at 100 µA via earlobes (Alpha-Stim AID device) Duration: 60 min/day for 3 weeks; as adjunct to a comprehensive inpatient pulmonary rehabilitation programme The rehabilitation programme consisted of: cycle endurance training (10–20 min, 60–70% peak workload, 5×/week), resistance training (3 sets, 15–20 reps, 5×/week), calisthenics and activities of daily living training (4–5×/week), Nordic walking or aqua fitness (2×/week), respiratory physiotherapy (2–4×/week), relaxation training, patient education, psychological support, occupational therapy, and nutritional counselling	Sham CES device (identical appearance, no active current) + identical comprehensive inpatient rehabilitation programme	Primary: Anxiety (Beck Anxiety Inventory, BAI) Secondary: Depression: PHQ-9 Fatigue: Fatigue Assessment Scale (FAS) Sleep quality: Pittsburgh Sleep Quality Index (PSQI); daily diary (10-point Likert scale)	Both groups improved significantly in anxiety (CES: *p* = 0.037; sham: *p* = 0.018) and depression (CES: *p* = 0.046; sham: *p* = 0.001) over the 3-week rehabilitation period, but no significant between-group difference was detected for BAI (*p* = 0.82) or PHQ-9 (*p* = 0.29)
Kuut et al., 2023	Adults with severe fatigue 3–12 months after COVID-19 *n* = 114 IG: *n* = 57 (79% F) Mean age: 45.7 ± 12.4 y/o CG: *n* = 57 (67% F) Mean age: 46.0 ± 12.9 y/o	Psychological/behavioural therapy: individualized CBT for severe post-COVID fatigue (multicomponent, blended online + optional in-person) CBT “Fit after COVID” targeting 7 perpetuating factors (sleep–wake, beliefs, activity regulation, social support, processing COVID-19, fears/worries, pain coping) Duration: 17 weeks; Total therapist–patient contacts: 11.8 ± 3.5 per participant (mean)	Care as usual: no access to CBT platform; unrestricted routine care	Primary: Fatigue (CIS-fatigue) Secondary – continuous: Quality of life (SF-36) Functional impairment (WSAS) Somatic symptoms (PHQ-15) Cognition (CIS-concentration) Secondary – dichotomous: Fatigue response (CIS-fatigue < 35) Fatigue response (CIS-fatigue < 35 + reliable change) Fatigue status (not chronically fatigued)	Patients receiving CBT had significantly lower fatigue severity than those receiving care as usual across follow-up assessments, with an overall mean between-group difference of −8.8 points (95% CI −11.9 to −5.8; *p* < 0.001; Cohen’s *d* = 0.69). The effect was present both directly post-treatment (T1: −9.3, 95% CI −13.3 to −5.3; *p* < 0.001) and at 6-month follow-up (T2: −8.4, 95% CI −13.1 to −3.7; *p* < 0.001).
Maritescu et al., 2024	Adults with PCC (moderate–severe dyspnoea and fatigue) *n* = 61 PR+PMR IG = 31 (25.81% F) Mean age: 64.67 ± 4.44 y/o PR-only CG = 30 (26.67% F) Mean age : 66.50 ± 5.19 y/o	Pulmonary rehabilitation combined with progressive muscle relaxation (PR+PMR) Duration: 21 days; aerobic exercise 5 days/week; strength training 3 days/week (non-consecutive); breathing exercises daily; PMR 20 min/daily Aerobic training: walking, cycling, treadmill exercise Strength training: bodyweight and resistance band; ~50%→1RM progressively to 70% 1RM Breathing: diaphragmatic, pursed-lip, lung expansion Intensity monitored via Borg RPE (target 4–6 for aerobic); progressive load increases based on tolerance	Active comparator: pulmonary rehabilitation only (PR), without progressive muscle relaxation; identical aerobic, strength, and breathing exercise components	Exercise capacity: 6MWT Pulmonary function: FVC (L, %pred), FEV_1_ (L, %pred), FEV_1_/FVC Psychological well-being: GHQ-12 Depression: PHQ-9 Anxiety: GAD-7 Sleep quality: PSQI	Addition of PMR significantly improved psychological outcomes beyond pulmonary rehabilitation alone. GHQ-12 significantly favoired the IG (12.16 ± 1.59 vs 17.06 ± 2.76; *p* < 0.001). PHQ-9 was significantly lower in the IG (median 7 vs 10.50; *p* < 0.001), with the IG achieving clinically significant reduction (≥ 5 points) GAD-7 significantly favoured the IG (4.96 ± 1.40 vs 8.23 ± 2.02; *p* < 0.001), with a reduction exceeding the MCID of 4 points. PSQI significantly favoured the IG (median 4 vs 7; *p* < 0.001), with the IG crossing the threshold of ≤5 (“good sleep”). 6MWT improved significantly within both groups, with no significant between-group difference (*p* = 0.171). Pulmonary function improved in both groups without significant between-group comparisons reported
Mooren J M et al., 2023	*n* = 110 IT: *n* = 45 (35.6% F) Mean age: 50.5 ± 10.1 y/o CT: *n* = 65 (40.0% F) Mean age: 48.4 ± 12.8 y/o CAD comparison cohort: *n* = 96 (25% F) Mean age: 50.3 ± 6.6 y/o	Interval training (IT) on a cycle ergometer Aerobic endurance training (cycle-ergometer) as part of comprehensive inpatient medical rehabilitation; both groups additionally received standard care (individualized physio/exercise therapies (e.g., group exercise, aqua fitness, terrain walking, circuit training) Duration: 4–6 weeks; 3–5 sessions/week of cycle-ergometer training; each session 18 min Intensity: IT: 60% W for 100 s/30% W for 48 s (isocaloric with CT) CT: constant 50% of maximal workload HR targets by Karvonen formula; loads adjusted by perceived exertion; HR monitored each session	Continuous training (CT) on a cycle ergometer + same rehab programme	Primary: Maximal exercise capacity (VO_2_peak) Secondary: Submaximal exercise capacity (VO_2_ at VT_1_) Fatigue (MFI-20) Quality of life (SF-36; WHO-5) Anxiety and depression (HADS) Work ability (WAI)	VO_2_peak increased in both groups over 4–6 weeks of inpatient rehabilitation, with no significant difference between training modalities. In the interval training group, VO_2_peak changed from 18.5 ± 4.3 to 18.7 ± 4.4 mL·min^–^¹·kg^–^¹ (Δ +0.2 ± 2.9), while in the continuous training group it increased from 18.2 ± 4.5 to 19.1 ± 5.2 mL·min^–^¹·kg^–^¹ (Δ +1.3 ± 2.9); the between-group difference was not significant (*p* = 0.157)
Navas-Otero et al., 2024	Adults with PCC *n* = 54 IG: *n* = 27 (76.5%F) Mean age: 44.06 ± 11.09 y/o CG: *n* = 27 (84.6%F) Mean age: 43.10 ± 9.23 y/o	Dual approach: 1. Symptom monitoring/recognition: Cognitive restructuring, disease education, relaxation 2. Functional adaptation: Goal setting, problem-solving, activity scheduling, posture training, motor imagery Duration: 6 weeks; 2 sessions/week 45–60 min each Intensity: No exercise dosing	Usual care + informational leaflet on long COVID symptoms	Quality of life (EQ-5D-5L) Disability (WHODAS 2.0) Functional impairment (WSAS) *No differentiation of primary/secondary outcomes*	Lifestyle-adjustment pro-gramme led to significant improvements in health-related quality of life, with favuorable between-group differences for EQ-5D self-care (*p* = 0.004) and anxiety/depression (*p* = 0.009), and reduced disability on WHODAS self-care (*p* = 0.014). Within the intervention group, multiple EQ-5D domains and EQ-VAS improved from baseline, alongside significant reductions in WHODAS total score and key subdomains (communication, mobility, self-care, and home life). Functional impairment (WSAS) also decreased in the intervention group, with no significant changes observed in controls
Omarova et al., 2023	Inpatients with PCC *n* = 160 IG: *n* = 80 (82.5% F) Mean age: 62.4 ± 4.54 y/o CG: *n* = 80 (68.8% F) Mean age: 60.2 ± 3.91 y/o	CRM with acupuncture CRM: respiratory gymnastics, massage, myorelaxation, physical therapy, speleotherapy, exercise equipment, aerosol therapy, oxygen cocktail, magnetotherapy, amplipulse, ultrawave frequencies, ultrasound therapy, ultraviolet irradiation, shungite therapy, inhalation, and outdoor walks Duration: 10–14 days; Acupuncture once daily for 10–14 sessions, 30 min/session Intensity: Acupuncture dose: full-body dry acupuncture at 10 points (SP6, ST36, LU5, LI4—bilateral; GV20—uni; +1 symptom-specific point [e.g., LU7/HT7/GB20/SP10/TE23]); ~11 needles (0.3×25–50 mm); 10 mm depth; manual stimulation to Deqi (bidirectional rotation 18 s) No aerobic/strength % targets reported	CRM only	Primary: Dyspnoea (MDS; mBorg) Physical function (6MWT) Activities of daily living (BI)	Compared with complex rehabilitation alone, rehabilitation plus acupuncture showed significantly greater improvements across all assessed outcomes, including dyspnoea (mMRC), perceived exertion (Borg), functional independence (Barthel Index), and exercise tolerance (6-minute walk test), whereas rehabilitation alone improved only dyspnoea and Borg scores; between-group differences favoured the acupuncture group (ANCOVA, all *p* < 0.001)
Ovejero et al., 2025	Adults with PCC (> 97% with symptoms persisting > 1 year) *n* = 98 IG = 51 (88.2% F) Mean age: 47.84 ± 9.93 y/o CG = 47 (83.0% F) Mean age: 48.95 ± 8.43 y/o	Balneotherapy (BT) combined with aquatic exercises in natural thermal mineral water (35°C). Duration: 4 weeks; 3 sessions/week on alternate days; 12 sessions; ~2 hours/session Session components: inhalation of thermal water vapor (10 min, alternating nose/mouth), circular shower (10 min, fine jet ankles-to-neck), structured aquatic exercise programme (15 min), and relaxation in whirlpool bath (15–20 min) Session structure alternated between full (pool + inhalation + shower + aquatic exercise) and abbreviated (pool + inhalation + shower) formats	Usual care	Functional status: PCFS Dyspnoea: mMRC scale Health-related QoL: SF-36 (8 domains) Sleep quality: PSQI Anxiety/Depression: HADS Cognitive function: MFE-30 Pain: VAS	Balneotherapy significantly improved fatigue (SF-36 Energy/Fatigue: β = −17.45; 95% CI: −24.23 to −10.66; *p* < 0.0001), pain (SF-36 Pain: β = −21.63; *p* < 0.0001; VAS: β = 1.625; *p* = 0.026), and social functioning (SF-36: β = −16.51; *p* < 0.0001) compared with usual care; fatigue and social functioning effects were sustained at 1-month follow-up Sleep quality (PSQI: β = 2.641; *p* = 0.003) and anxiety (HADS-A: β = 1.72; *p* = 0.023) improved significantly post-intervention but were not sustained at follow-up. SF-36 role limitations due to physical health (β = −10.63; *p* = 0.026) were also significantly improved. No significant between-group differences were observed for physical functioning, mMRC, PCFS, cognitive function (MFE-30), depression, or general health
Polat et al., 2025	Adults with PCC *n* = 74 VRG = 37 (81.1% F) Mean age: 43.3 ± 8.4 y/o CTG = 37 (75.7% F) Mean age: 45.3 ± 8.6 y/o	Semi-immersive, motion-controlled video game-based virtual reality (VR) exercise via Microsoft Xbox One Kinect console and Kinect V2 infrared camera Duration: 8 weeks; 3 sessions/week; 30–45 min/session Participants performed whole-body sensorimotor and plyometric movements (jumping, shooting, limb/spine movements) to control an avatar in motion-controlled games (tennis, bowling, soccer, target shooting, climbing, jet ski racing) Task difficulty progressed weekly based on ability to complete tasks and defeat the virtual opponent	Active comparator: structured conventional exercise programme consisting of flexibility training, aerobic bicycle ergometer (60%→75% HRmax, +5%/2 weeks), resistance training with dumbbells/sandbags (50%→70% 1RM), and neuromuscular balance exercises (Bosu ball/balance board). Same frequency and duration	Primary: Pain intensity (VAS) Secondary: Fatigue: FSS Anxiety/Depression: HADS (HADS-A, HADS-D) Health-related QoL: SF-12 (PCS, MCS) Functional exercise capacity: 6MWT	VR exercise produced significantly greater pain reduction compared with conventional exercise (time×group: F(1,59.4) = 56.31, *p* < 0.001; partial η² = 0.487; between-group difference −1.37, 95% CI: −1.73 to −1.00). VAS scores changed from 6.4 ± 1.3 to 4.2 ± 1.1 in VRG vs 6.1 ± 1.2 to 5.3 ± 1.2 in CTG; MCID of ≥ 30% achieved by 76% vs 54%
Rutkowski et al., 2023	Adults with PCC *n* = 32 IG: *n* = 18 (61.11% F) Mean age: 59.22 ± 3.89 y/o CG: *n* = 14 (64.29% F) Mean age: 56.00 ± 6.57 y/o	Comprehensive CPR: cycle-ergometer endurance, breathing exercises, general fitness, resistance training, and relaxation + VR-PR: head-mounted display during cycling (Virtual Park) and VR-guided relaxation (TierOne) Duration: 3 weeks; 5×/week inpatient programme; cycle-ergometer twice daily, all other components once daily Intensity: Training HR set from 6MWT heart-rate (models A–D): ~90%/80%/70% of peak 6MWT HR or +20–30% over rest (model-dependent) Work-rate progressed by ± 5 W based on prior HR attainment; cadence 50–70 RPM Monitoring: HR, SpO_2_, Borg dyspnoea/fatigue each session	Multidisciplinary programme as intervention, delivered in a traditional (non-VR) format	Physical function (6MWT) Dyspnoea (mBorg) Lung function (FEV_1_; FVC; FEV_1_/VC; TLC) Psychological stress (PSS-10) *No differentiation of primary/secondary outcomes*	Both VR-based and conventional inpatient rehabilitation resulted in significant improvements in functional exercise capacity, with increases in 6-minute walk distance (+56.9 m and +39.2 m, respectively; both *p* < 0.001), and reductions in exertional dyspnoea following the 6MWT. Stress levels (PSS-10) decreased significantly in both groups, while no statistically significant between-group differences were observed for any outcome. Spirometric measures remained unchanged in both groups
Santana et al., 2023	Adults with PCC-related fatigue *n* = 70 IG: *n* = 35 (69% F) Mean age: 51.63 ± 15.87 y/o CG: *n* = 35 (60% F) Mean age: 54.46 ± 19.01 y/o	HD-tDCS combined with a rehabilitation programme: Submaximal exercise Gradual stretching Breathing exercises Resistance training Patient education for self-management and coping Duration: 5 weeks, 2 sessions/week Intensity: Stimulation: 4×1 HD-tDCS targeting left M1, 3 mA, 30 min, 30-s ramp up/down Rehab during each session: submaximal exercise at RPE 9–11 (very light–light) with stretching, breathing, and resistance work; progression guided by Borg breathlessness/RPE and symptoms	Sham HD-tDCS (identical setup, brief ramp only) + the same rehabilitation programme	Primary: Fatigue (MFIS: total; cognitive; psychosocial; physical subscales) Secondary: Anxiety (HAM-A) Quality of life (WHOQOL-BREF) Pain (McGill Pain Questionnaire)	After 5 weeks, active HD-tDCS plus rehabilitation produced a significantly greater reduction in fatigue than sham stimulation. MFIS total scores decreased by −22.11 points in the active group vs −10.34 points in the sham group, corresponding to a mean between-group difference of −14.03 points (95% CI −20.28 to −7.78; *p* < 0.001; effect size 1.2)
Sick et al., 2025	Adults with PCC *n* = 42 ED = 14 (85.7% F) Mean age: 41.8 ± 11.8 y/o CT = 14 (71.4% F) Mean age: 41.6 ± 14.7 y/o CG = 14 (78.6% F) Mean age: 40.3 ± 10.8 y/o	(a) Supervised endurance training (ED): aerobic exercise on cycle ergometer, elliptical, or treadmill; intensity prescribed relative to VT1/VT2 from CPET; 30–60 min/session (b) Supervised concurrent training (CT): resistance training (leg press, leg curl, chest press, horizontal row; 80% 5RM; RPE 14–18) followed by aerobic exercise (20–40 min) Both programmes: 12 weeks; 3 sessions/week on non-consecutive days Volume increased progressively (≤5 min/session/week if tolerated). SpO_2_ monitored at least once/session. Pre-session VAS ratings of well-being, energy, sleep, pain, and symptom severity recorded	Usual care	Primary: VO_2_peak (CPET) Secondary: Strength: Isometric leg press (N), handgrip (kg) Heart rate variability: RMSSD, SDNN Symptom count: NICE Symptom Checklist Fatigue: FSS Dyspnoea: mMRC QoL: SF-36 (physical functioning, energy/fatigue, total score); Cognitrone concentration test	Both exercise groups significantly improved VO_2_peak compared with usual care (time×group: *p* = 0.002, ηp² = 0.267): ED +3.9 mL/min/kg (*p*≤0.001, g = 1.75); CT +3.2 mL/min/kg (*p* = 0.001, g = 1.02); CG non-significant. No superiority of either exercise modality was established
Sirydakis et al., 2024	Adults with PCC and chronic post-COVID fatigue *n* = 39 IG = 21 (47.6% F) Mean age: 47.61 ± 12.13 y/o CG = 18 (44.4% F) Mean age: 54.05 ± 11.43 y/o	Supervised, face-to-face multicomponent physical training at a university rehabilitation centre Duration: 11 weeks (1-week familiarization + 10 weeks); 2 sessions/week (Mondays and Fridays); 60 min/session; 22 total training sessions Session structure: warm-up (5 min), balance training (10 min), aerobic interval treadmill exercise (25 min), resistance training (15–20 min), stretching (5 min) Aerobic and strength components alternated weekly across 2 mesocycles (weeks 1–6 and 7–11) Aerobic: interval treadmill walking/running; Mesocycle 1: 5×3 min or 3×5 min; Mesocycle 2: 5×4 min or 4×5 min Resistance: 5 exercises targeting major muscle groups (sit-to-stand, bench press, step up/down, band row); 2→3 sets ×10–15 reps Intensity: RPE 12–13 (Borg scale) for both aerobic and resistance components. SpO_2_, HR, BP, and capillary blood glucose monitored throughout sessions	Education-based control: single in-person session at baseline with physical activity recommendations based on national guidelines, plus one follow-up telephone contact at week 6	Primary: Six-Minute Walk Test (6MWT) Secondary: Health-related QoL: EQ-5D-5L (overall score) Health perception: EQ-5D VAS Sleep quality: Pittsburgh Sleep Quality Index – Brazilian version (PSQI-BR) Depressive symptoms: PHQ-9	6MWT and EQ-5D VAS did not show significant changes in either group No significant 6MWT between-group effect was found, suggesting functional exercise capacity gains were not demonstrated within the study period
Volckaerts et al., 2025	Adults with PCC *n* = 76 IG = 39 (61.5% F) Mean age: 50.7 12.6 y/o CG = 37 (67.6% F) Mean age: 48.1 ± 12.6 y/o	Standardized, personalized, stepwise pulmonary rehabilitation (PR) in primary care delivered as individual face-to-face physiotherapy sessions Duration: 12 weeks; 3×30-min sessions/week. Five progressive phases: (1) education about long COVID and lifestyle, (2) endurance training, (3) strength training, (4) inspiratory muscle training (Philips Respironics Threshold IMT), (5) functional breathing exercises Additional modalities: relaxation (54%), mucus clearance (21%) Intensity monitored via Perceived Training Recovery (PTR) and Perceived Training Intensity (PTI) scores combined into a Total Training Score (TTS) at each session, guiding progression to the next phase, maintenance, or step-down Designed to prevent post-exertional malaise (PEM)	Usual care	Primary: 6MWD Secondary: Fatigue: CIS-fatigue Health status: CAT questionnaire Health-related QoL: EQ-5D-5L (utility index, VAS) Activity: daily step counts (accelerometry) Exploratory: PCFS, HADS, mMRC, Nijmegen questionnaire, HGS, MIP, MEP, spirometry	Pulmonary rehabilitation significantly improved functional exercise capacity. Between-group treatment effect: +39 m (95% CI: 18–59; *p* < 0.001) at week 12, sustained at week 24 (+47 m; 95% CI: 26–68; *p* < 0.001; time×group: *p* < 0.0001). IG participants were significantly more likely to achieve clinically meaningful improvement (OR 5.7, 95% CI: 2.0–16.1; *p* = 0.001)
Zha et al., 2024	Adults with PCC *n* = 95 IG: *n* = 47 (70.2% F) Mean age: 57.5 y/o CG: *n* = 48 (60.4% F) Mean age: 59.5 y/o	Intermittent hypoxia exposure (normobaric) as an add-on to routine therapy (bronchodilators, nebulized corticosteroid/anticholinergic as indicated). No exercise training Duration: approx. 10 days, daily sessions Intensity: IHE: 5 min hypoxia (FiO_2_ 10–12%) ↔ 5 min room air, ×5 cycles per session; hypoxia titrated to maintain SpO_2_ 75–85% (abort if < 75% or discomfort; give O_2_ to 95%)	Normoxia exposure (compressed air 50 min with matched flows) + routine therapy	Primary: Physical function (6MWD) Lung function (VT; FVC; FVC %pred; FEV_1_; FEV_1_ %pred; FEV_1_/FVC) Dyspnoea (Borg; mMRC) Fatigue (FAS; CFQ-11) Secondary: Safety (adverse events related to interventions) Patient-reported symptom improvement (dyspnoea; fatigue: no/slight vs obvious improvement) Intervention dose–response (≥ 10 days vs < 10 days of IHE)	Intermittent hypoxia exposure (IHE) resulted in significantly greater improvements than normoxia exposure (NE) across all prespecified primary outcomes. Physical function (6MWD) increased by +47.0 m (IQR 30.0–61.0) with IHE vs +23.5 m (IQR 11.5–33.0) with NE (*p* = 0.001). Lung function improved significantly in the IHE group compared with NE for tidal volume (VT), FVC, FVC %pred, FEV_1_, FEV_1_ %pred, and FEV_1_/FVC (all *p* < 0.05). Dyspnoea scores improved more with IHE than NE on both the Borg scale and mMRC (all *p* < 0.05). Fatigue decreased significantly in the IHE group compared with NE on both FAS and CFQ-11 (all *p* = 0.001)
Zilberman-Itskovich et al., 2022	Adults with PCC *n* = 73 IG: *n* = 37 (51.35% F) Mean age: 48.4 ± 10.6 y/o CG: *n* = 36 (69.4% F) Mean age: 47.8 ± 8.5 y/o	Hyperbaric oxygen therapy Duration: 8 weeks; 40 sessions (5×/week); 90 min/session Intensity: HBOT protocol: 2.0 ATA, 100% O_2_ by mask, 90 min with 5-min air breaks every 20 min; compression/decompression 1.0 m/min	Sham: 1.03 ATA, 21% O_2_, 90 min, with brief pressurization to 1.2 ATA for 5 min + decompression over 5 min to mimic sensations	Primary: Cognitive function (NeuroTrax computerized battery) Secondary: Brain imaging (MRI: T2-weighted; 3D FLAIR; SWI; pre/post-contrast MPRAGE 3D T1; DSC perfusion maps; DTI: FA & MD maps) Quality of life (SF-36) Sleep quality (PSQI) Psychological distress (BSI-18) Pain (BPI) Olfaction (Sniffin’ Sticks Test) Taste (Taste Strip Tes) Lung function (FVC; FEV_1_; VC; TLC; RV; DLCO) Blood tests (CBC, chemistry, inflammatory markers) Safety (adverse events)	HBOT led to a significantly greater improvement in global cognitive function compared with sham, with a significant group-by-time interaction (Cohen’s *d* = 0.495; *p* = 0.038). Global cognitive scores increased from 98.3 ± 11.1 to 104.1 ± 7.2 in the HBOT group (*p* < 0.001) vs 98.9 ± 8.5 to 101.3 ± 8.9 in controls (*p* = 0.010), favouring HBOT

ACE-III: Addenbrooke’s Cognitive Examination III; ANCOVA: analysis of covariance; APSI: Anterior-Posterior Stability Index; AT: anaerobic threshold; ATA: atmospheres absolute; BAI: Beck Anxiety Inventory; BDI: Beck Depression Inventory; BFI: Brief Fatigue Inventory; BI: Barthel Index; BP: blood pressure; BPI: Brief Pain Inventory; BRIEF-A: Behavior Rating Inventory of Executive Function – Adult Version; BRI: Behavioral Regulation Index; BSI-18: Brief Symptom Inventory-18; C19-YRSm: modified COVID-19 Yorkshire Rehabilitation Scale; CAT: COPD Assessment Test; CBC: complete blood count; CBT: cognitive behavioural therapy; CES: cranial electrotherapy stimulation; CFQ-11: Chalder Fatigue Questionnaire-11; CFS: Chalder Fatigue Scale; CG: control group; CI: confidence interval; CIS-fatigue: Checklist Individual Strength – Fatigue Subscale; CPET: cardiopulmonary exercise testing; CPRP: cardiopulmonary rehabilitation programme; CRM: complex rehabilitation measures; CST: 30-Second Chair-Stand Test; CT: concurrent training (also used as continuous training; Mooren et al.); CTG: conventional training group; DLCO: diffusing capacity of the lungs for carbon monoxide; DSB: digit span backward; DSC: dynamic susceptibility contrast; DSQ-PEM: DePaul Symptom Questionnaire – Post-Exertional Malaise; DTI: diffusion tensor imaging; ED: endurance training group; EEG: electroencephalography; EPAP: expiratory positive airway pressure; EQ-5D-5L: EuroQol 5-Dimension 5-Level Questionnaire; F: female; FA: fractional anisotropy; FAS: Fatigue Assessment Scale; FEV_1_: forced expiratory volume in 1 second; FiO_2_: fraction of inspired oxygen; FLAIR: fluid-attenuated inversion recovery; Fmax: maximum handgrip force; Fmean: mean handgrip force; FRI: Fall Risk Index; FSS: Fatigue Severity Scale; FVC: forced vital capacity; GAD-7: Generalized Anxiety Disorder 7-Item Scale; GEC: global executive composite; GHQ-12: General Health Questionnaire-12; HADS: Hospital Anxiety and Depression Scale; HADS-A: Hospital Anxiety and Depression Scale – Anxiety Subscale; HADS-D: Hospital Anxiety and Depression Scale – Depression Subscale; HAM-A: Hamilton Anxiety Rating Scale; HBOT: hyperbaric oxygen therapy; HD-tDCS: high-definition transcranial direct current stimulation; HGS: handgrip strength; HIIT: high-intensity interval training; HR: heart rate; HRmax: maximum heart rate; HRR: heart rate reserve; IHE: intermittent hypoxia exposure; IG: intervention group; IMT: inspiratory muscle training; IQR: interquartile range; ISI: Insomnia Severity Index; IT: interval training; LICT: low-intensity continuous training; M1: primary motor cortex; MathProc: Math Processing Test; mBorg: Modified Borg Scale; MCS: mental component summary; MCID: minimal clinically important difference; MD: mean diffusivity; MDS: Modified Dyspnea Scale; MEP: maximum expiratory pressure; MFE-30: Memory Failures in Everyday Life Questionnaire (30-item); MFIS (MFIS-21): Modified Fatigue Impact Scale (21-item version); MFI-20: Multidimensional Fatigue Inventory-20; MI: Metacognition Index; MIIT: moderate-intensity interval training; MIP: maximum inspiratory pressure; MIVT: moderate-intensity variable training; MLSI: Medial-Lateral Stability Index; mMRC: Modified Medical Research Council Dyspnea Scale; MMQ: Multifactorial Memory Questionnaire; MoCA: Montreal Cognitive Assessment; MPRAGE: magnetization-prepared rapid gradient echo; MRC: Medical Research Council Dyspnea Scale; MRI: magnetic resonance imaging; NE: normoxia exposure; OMNI-RES: OMNI Resistance Exercise Scale; OQ-45: Outcome Questionnaire-45; OR: odds ratio; OSI: Overall Stability Index; OUES: oxygen uptake efficiency slope; PCC: post-COVID-19 condition; PCFS: Post-COVID Functional Status Scale; PCS: physical component summary; PEM: post-exertional malaise; PEEP: positive end-expiratory pressure; PHQ-9: Patient Health Questionnaire-9; PHQ-15: Patient Health Questionnaire-15; PIM: peak inspiratory muscle pressure; PMR: progressive muscle relaxation; PR: pulmonary rehabilitation; PSM-25: Psychological Stress Measure-25; PSS (PSS-10): Perceived Stress Scale (10-item version); PSQI: Pittsburgh Sleep Quality Index; PSQI-BR: Pittsburgh Sleep Quality Index – Brazilian Version; PTI: perceived training intensity; PTR: Perceived Training Recovery; Ptrails: Trail-Making Test (digital neurocognitive version); QLSI: Quality of Life Systemic Inventory; RAND-36: RAND 36-Item Health Survey; REA: simple reaction time test; RHI: Reactive Hyperaemia Index; RM: respiratory muscle training; RMSSD: root mean square of successive differences; RPE: rating of perceived exertion; RV: residual volume; SDNN: standard deviation of normal-to-normal RR intervals; SF-12: 12-Item Short Form Health Survey; SF-36: 36-Item Short Form Health Survey; SHAI: Short Health Anxiety Inventory; SpO_2_: peripheral oxygen saturation; STAI: State-Trait Anxiety Inventory; SWI: susceptibility-weighted imaging; THR: target heart rate; TLC: total lung capacity; TTS: total training score; TUG: Timed Up and Go Test; VAS: Visual Analogue Scale; VC: vital capacity; VE/VCO_2_: ventilatory equivalent for carbon dioxide; VO_2_ at AT: oxygen uptake at anaerobic threshold; VO_2_max: maximum oxygen uptake; VO_2_peak: peak oxygen uptake; VR: virtual reality; VRG: Virtual Reality Group; VR-PR: virtual reality-based pulmonary rehabilitation; VT: tidal volume; VT_1_: ventilatory threshold 1; VT_2_: ventilatory threshold 2; WAI: Work Ability Index; WHO: World Health Organization; WHO-5: World Health Organization-Five Well-Being Index; WHODAS 2.0: World Health Organization Disability Assessment Schedule 2.0; WHOQOL-BREF: World Health Organization Quality of Life – Brief Version; WPAI: Work Productivity and Activity Impairment Questionnaire; WR: work rate; WSAS: Work and Social Adjustment Scale; y/o: years old; 1RM: one-repetition maximum; 5STS: Five Times Sit-to-Stand Test; 6MWD: Six-Minute Walk Distance; 6MWT: Six-Minute Walk Test; 6MWW: Six-Minute Walk Work.

### Description of the population

A total of 1,998 adults with PCC were enrolled in the 27 randomized controlled trials. with a median of 63 participants per trial; trial sizes varied from 16 (17) to 160 (18) participants. The age distribution was primarily middle-aged, with the median of the study-level reported means being 48.81 years and reported group-level mean ages ranging from 31.0 ± 11.85 years (19) to 74.25 ± 7.31 years (20). The included trials had a median female proportion of 66.1%. The mean duration of symptoms before enrolment ranged from 3.2 months (21) to 25.6 months (22); the median of the reported mean or median durations was 10.7 months. The duration of the received interventions ranged from 10 days (23) to 17 weeks (24), with a median of 6 weeks.

### Description of the outcomes

The included studies assessed a broad spectrum of clinical, functional, and physiological outcomes, reflecting the multisystemic nature of PCC. Across the review, these parameters were categorized into 4 primary domains: patient-reported symptom severity, health-related quality of life (HRQoL), mental health status, and objective functional or physiological capacity.

The most frequently evaluated domains focused on symptom burden and quality of life, utilizing various standardized patient-reported outcome measures (PROMs). Fatigue was a primary focus in nearly all trials, most commonly measured via the Fatigue Severity Scale (FSS), the Chalder Fatigue Scale (CFS/CFQ-11), the Modified Fatigue Impact Scale (MFIS), or the Checklist Individual Strength (CIS-fatigue) (25–34). Dyspnoea was similarly prioritized, with studies primarily using the modified Medical Research Council (mMRC) scale and the Borg Dyspnea Scale (18, 26, 29, 31, 33, 35, 36).

Health-related quality of life was quantified using diverse instruments, most notably the Short Form-36 (SF-36) or its abbreviated SF-12 and RAND-36 versions, as well as the EuroQol-5D (EQ-5D-3L/EQ-5D-5L) and the WHOQOL-BREF (20–22, 26–30, 32, 36–42). Mental health status, specifically anxiety and depression, was widely assessed through the Hospital Anxiety and Depression Scale (HADS), the Patient Health Questionnaire-9 (PHQ-9), and the Generalized Anxiety Disorder-7 (GAD-7) (19, 21, 26, 30, 41, 43, 44). Physiological and objective functional outcomes were consistently reported to quantify exercise tolerance and physical recovery. The 6-Minute Walk Test (6MWT) served as the predominant measure of submaximal functional exercise capacity (18, 22, 25, 27, 30, 31, 33, 35–37). For a more detailed assessment of cardiorespiratory fitness, several studies utilized maximal cardiopulmonary exercise testing (CPET) to determine peak oxygen consumption (VO_2peak_) and ventilatory thresholds (21, 29, 36, 45).

Muscle strength was objectively evaluated using handgrip dynamometry and one-repetition maximum (1RM) tests for the upper and lower limbs (26, 29, 30, 32, 33, 44). Additional physiological assessments included spirometry (FEV1, FVC), heart rate variability (HRV), and body composition analysis (21, 22, 26, 29, 31, 33, 35, 36, 41).

Less frequently investigated domains included cognitive function, which was screened using the Montreal Cognitive Assessment (MoCA) or more comprehensive computerized neuro-psychometric batteries (25, 38, 39, 42). A minority of studies specifically screened for post-exertional malaise (PEM) using the DePaul Symptom Questionnaire (DSQ-PEM) or monitored indices of autonomic function and brain MRI perfusion (22, 30, 32, 42). Overall, outcome reporting demonstrated considerable heterogeneity regarding the selection of primary instruments and the specific subscales utilized across the different trials.

### Risk of bias in studies

We assessed all 27 included randomized controlled trials using the Cochrane RoB 2 tool across 5 domains (Fig. 2). Overall, 5 of 27 trials (18.5%) were judged at low risk of bias (22, 28, 31, 33, 39), 17 of 27 (63.0%) raised some concerns (19, 21, 25–27, 29, 30, 32, 36–38, 40–44, 46), and 5 of 27 (18.5%) were rated at high risk of bias (18, 20, 34, 35, 45). Randomization (D1) and selection of reported results (D5) were generally robust, with no study rated at high risk in either domain. Deviations from intended interventions (D2) and measurement of the outcome (D4) represented the main methodological concerns, with 2 studies rated at high risk in each domain (18, 20, 34, 35). Missing outcome data (D3) also contributed to overall uncertainty, with 1 study judged at high risk and several studies raising some concerns. High-risk judgements were mainly related to lack of blinding, reliance on self-reported or otherwise unblinded outcome assessment, and attrition-related missing data.

**Fig. 2:**
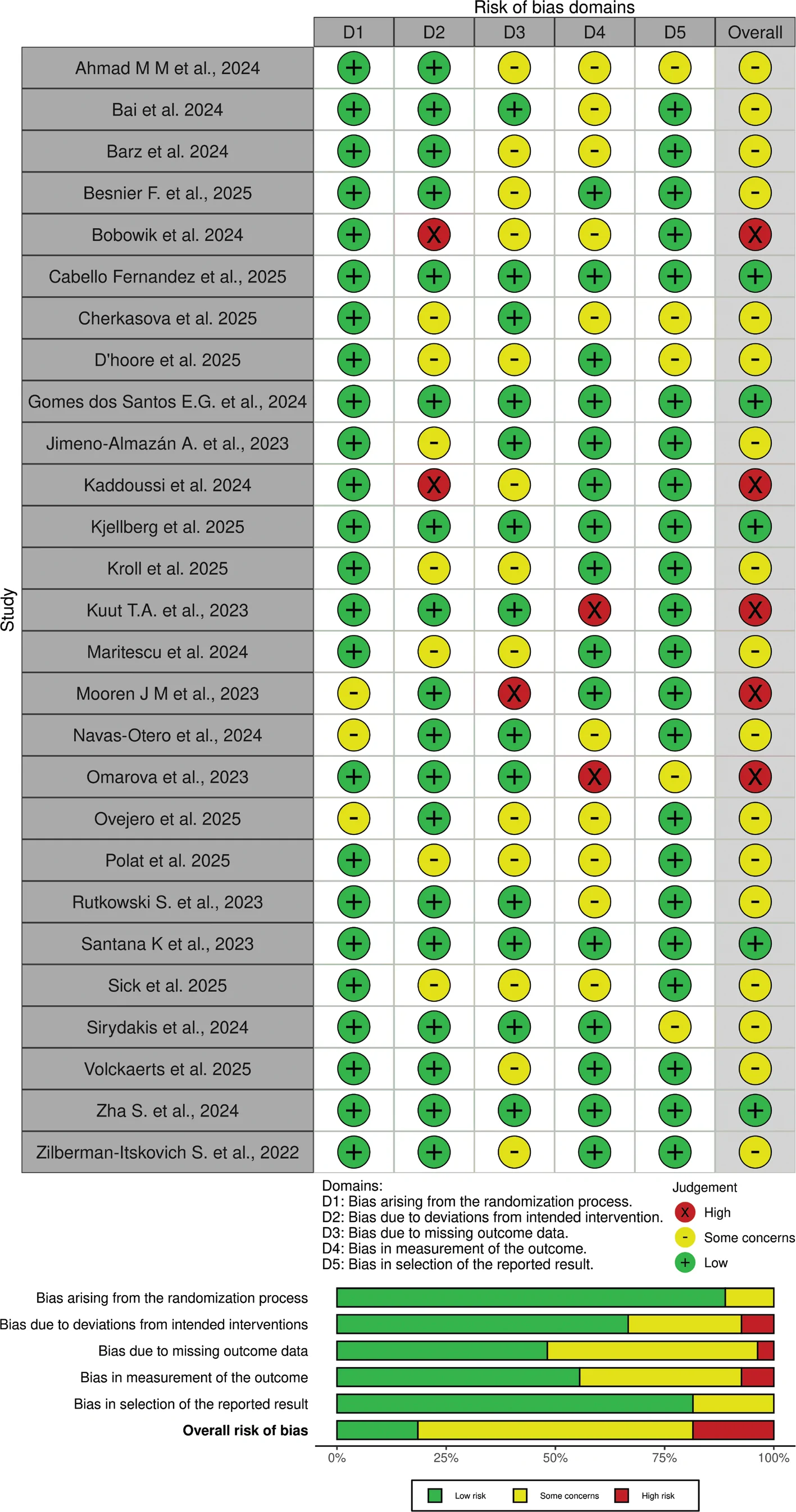
Risk of bias of randomized controlled trials (RoB 2.0).

### Description of the interventions

The interventions included in this systematic review were grouped into 7 categories. Cardiopulmonary rehabilitation (CPR) programmes consisted of supervised protocols integrating aerobic, resistance, and respiratory muscle training (26, 30, 33, 35, 36, 44, 46–48). Multimodal exercise involved programmes combining 2 or more training types within single sessions, such as semi-immersive virtual reality or concurrent resistance-endurance training (27, 29, 32, 37). Unimodal exercise focused on isolated training types, including cycle ergometry aerobic conditioning or targeted resistance protocols for postural stability (20, 21, 25, 45). Psychological and cognitive interventions provided structured behavioural and symptom management through approaches like cognitive-behavioural therapy (CBT) or lifestyle self-adjustment (34, 39, 40). Neuromodulation and neurofeedback utilized non-invasive electrical or neurophysiological stimuli to modulate brain states, including HD-tDCS, cranial electrotherapy stimulation, and EEG biofeedback (19, 28, 43). Oxygen-based therapies manipulated oxygen partial pressures or atmospheric pressure, featuring hyperbaric oxygen therapy or intermittent hypoxia exposure (22, 31, 38, 42). Finally, complementary and alternative interventions served as adjunct treatments targeting symptomatic relief via modalities such as dry-needling acupuncture and thermal balneotherapy with aquatic exercise (18, 41).

Of the 27 included trials, the control condition was usual care in 12 trials (20, 21, 26, 29, 30, 32, 34–37, 40, 41), placebo/sham in 7 trials (19, 22, 28, 31, 38, 42, 43), and an active comparator in 8 trials (18, 25, 27, 33, 39, 44–46).

*Cardiopulmonary rehabilitation*. Seven trials (reported in nine publications) included cardiopulmonary rehabilitation (CPR), which consisted of supervised protocols integrating aerobic, resistance, and respiratory muscle training (26, 30, 33, 35, 36, 44, 46–48). Compared with usual care, CPR consistently improved cardiorespiratory fitness (VO_2peak_), with mean between-group adjusted differences ranging from +2.1 to +2.7 mL/kg/min (36, 48). One trial reported a 5.7% increase in VO_2max_ following CPR, while control participants experienced a 0.8% decline (48). Functional exercise capacity, primarily assessed via the 6-minute walk test (6MWT), demonstrated a uniform pattern of improvement favouring CPR over usual care. Mean between-group increases in walking distance ranged from 39 m to 168 m (30, 33, 35, 36). In contrast, in a trial comparing CPR delivered with virtual reality against an active comparator receiving an identical CPR programme without virtual reality, both arms showed significant within-group improvements but no significant between-group difference in 6MWT distance (44, 46).

A consistent reduction in symptom burden was observed across trials comparing CPR with usual care, specifically for dyspnoea and fatigue. Reductions in the modified Medical Research Council (mMRC) scale significantly favoured CPR groups, with mean between-group differences ranging from 0.5 to 1.5 points (30, 33, 35). In 1 cohort, the proportion of participants with mMRC level 3 dyspnoea decreased from 75% to 5% following CPR (35). Fatigue scores also showed consistent between-group improvements across various scales, including the Fatigue Severity Scale and CIS-fatigue (26, 30). One trial comparing CPR against an active comparator receiving CPR plus progressive muscle relaxation (PMR) found that the PMR group achieved significantly greater reductions in fatigue and anxiety (44). Findings for pulmonary function (spirometric volumes) against usual care were consistently null for between-group differences in FEV_1_ and FVC (35, 36, 46, 48). However, respiratory muscle strength favoured CPR in some trials, with significant between-group improvements in maximal inspiratory and expiratory pressures (30). Outcomes for mental health and quality of life were heterogeneous when comparing CPR with usual care. While significant reductions in depression scores and improvements in physical quality-of-life domains (SF-12/SF-36) were frequently observed, effects on anxiety and global health-utility indices (EQ-5D-5L) were often null (26, 30, 36, 47, 48). Finally, neuropsychological test performance for executive function, memory, and processing speed showed no significant between-group differences following CPR compared with usual care (47).

*Multimodal exercise interventions*. Four RCTs evaluated distinct multimodal exercise modalities. Multimodal exercise interventions are defined as integrated physical training programmes that combine 2 distinct exercise modalities such as aerobic, resistance, balance, or flexibility training within a single session (27, 29, 32, 37). Primary outcomes for cardiorespiratory fitness and functional capacity consistently favoured exercise interventions over usual care. Cardiorespiratory fitness (VO2peak) demonstrated significant between-group improvements ranging from 3.2 to 3.9 mL/min/kg (29). While functional exercise capacity (6MWT) improved within all groups across trials, between-group differences were significant for endurance capacity (+27.1 steps) only when compared with non-exercising controls (27, 29, 32). Trials comparing active modalities with active comparators, such as motion-controlled virtual reality (VR) vs a conventional multicomponent training, reported no significant between-group differences in 6-minute walk distance (27, 29). Findings for muscular strength were heterogeneous; 1 trial reported significant between-group increases in handgrip strength vs usual care, while another found no significant differences against an active comparator (29, 32). Lower body strength consistently showed significant between-group improvements favouring exercise groups over usual care (29). Symptom burden, specifically fatigue and pain, demonstrated consistent favourability for multimodal exercise groups over usual care. Fatigue severity (FSS) showed significant between-group reductions, with score improvements ranging from 1.14 points (20.1%) to 4.19 points (27, 29, 32).

Pain intensity (VAS) significantly favoured VR-based multimodal exercise over a conventional active program, with a mean difference in change of –1.37 points (27). Quality of life (SF-12/SF-36) outcomes varied; significant between-group favourability was found in trials comparing exercise with usual care or physical activity recommendations (32, 37), but results were consistently null when comparing different active exercise modalities (27, 29). Secondary mental health outcomes showed significant within-group improvements, yet between-group effects were largely null for anxiety and total symptom counts against both usual care and active comparators (27, 29). One trial reported a significant between-group advantage for VR over an active comparator in reducing depressive symptoms (–1.16 difference in change) (27). Sleep quality significantly improved following multicomponent training compared with a usual care with physical activity recommendation (37). Finally, outcomes for post-exertional malaise (PEM), health perception, and concentration performance consistently demonstrated null between-group findings against usual care and active comparators (29, 32, 37).

*Unimodal exercise interventions*. Unimodal exercise interventions are defined as rehabilitation programmes that focus exclusively on a single training modality, such as isolated aerobic endurance conditioning or resistance-based protocols (20, 21, 25, 45). Primary outcomes for physical capacity consistently favoured exercise interventions over usual care, though comparisons between active modalities yielded null findings. Supervised aerobic training significantly improved peak oxygen uptake VO_2peak_, with a mean between-group increase of 4.64 mL/kg/min compared with a 1.06 mL/kg/min decline in a usual-care control group (21). Similarly, resistance training (RT) significantly enhanced postural stability and reduced fall-risk indices compared with usual care (20). In contrast, trials comparing different delivery modes of the same exercise type as an active comparator, such as interval vs continuous aerobic training or virtual reality (VR) vs conventional treadmill exercise, reported no significant between-group differences in VO_2peak_, workload, or walking capacity (25, 45). Findings for symptom resolution were heterogeneous. One aerobic trial reported that 67.8% of the intervention group experienced reduced or resolved symptoms compared with 16.7% of the usual-care control group (21). However, specialized active-comparator trials found that neither VR-simulated treadmill exercise nor interval-based training provided a significant between-group advantage for fatigue reduction over conventional continuous methods (25, 45). Secondary outcomes for quality of life (QoL) and mental health varied by intervention type. RT demonstrated significant between-group favourability over usual care in psychological and social relationship domains (20).

Aerobic training trials showed consistently null between-group effects for anxiety, depression, and overall health-related QoL (SF-12/SF-36) when compared with usual care or an active-comparator aerobic mode (21, 45). Across unimodal aerobic trials, cognitive function and sleep quality significantly improved within groups from baseline; however, no significant between-group differences were observed against usual care or an active comparator (21, 25). Furthermore, unimodal exercise did not produce significant between-group changes in pulmonary function (FEV1/FVC) or resting laboratory parameters compared with usual care (21, 45).

*Psychological interventions*. Psychological interventions, particularly cognitive-behavioural therapy (CBT) and lifestyle self-adjustment, consistently improved fatigue severity compared with usual care (34, 40). Between-group mean differences in fatigue scores favoured CBT by –8.4 to –9.3 points on the Checklist Individual Strength (CIS), representing a medium effect size (34). However, comparisons between active modalities, specifically cognitive vs affective psychoeducation as an active comparator, yielded null between-group findings, with both arms showing similar trajectories of improvement (39). Outcomes for subjective cognitive complaints showed no significant between-group differences when comparing different active psychoeducational approaches (39). In contrast, CBT demonstrated a significant between-group advantage over usual care in reducing concentration problems (mean difference –5.1 points) (34). Objective cognitive performance in attention and memory improved within-group across trials, but between-group effects remained largely heterogeneous or null against the active comparator (39).

Results for health-related quality of life (QoL) and functional disability consistently favoured intervention groups over usual care. Lifestyle adjustment programmes significantly improved QoL dimensions of self-care and anxiety/depression, alongside reductions in total disability scores (40). Physical and social functioning scores favoured CBT over usual care, with mean between-group differences of +7.1 and –6.6 points (34). One trial comparing active psychoeducational arms reported consistently null between-group effects for global QoL, sleep quality, and psychological distress (39).

*Alternative interventions*. Acupuncture and balneotherapy consistently improved fatigue and physical functioning compared with complex rehabilitation as an active comparator (acupuncture) and usual care (balneotherapy). Fatigue severity significantly improved, ranging from a 35% reduction in Borg scores following acupuncture to a significant increase in energy/fatigue subscales (Beta –17.45) after balneotherapy (18, 41). For physical capacity and disability, acupuncture demonstrated a 29.5% increase in walking distance and a 2.2% improvement in Barthel Index scores over the active-comparator complex rehabilitation regimen alone (18). Balneotherapy significantly reduced pain intensity (VAS Beta 1.62) and improved social and physical role limitations (41). Findings for respiratory symptoms were heterogeneous; acupuncture significantly reduced dyspnoea (mMRC 40.2% improvement), whereas balneotherapy demonstrated no significant between-group difference on the same scale (18, 41). Results for mental health and cognition were similarly varied. Balneotherapy significantly improved anxiety and sleep quality, but between-group effects for depression, global functional status (PCFS), and subjective memory failures were consistently null (41).

*Neuromodulation*. Three RCTs evaluated neuromodulation modalities: EEG α-rhythm biofeedback (BFB), high-definition transcranial direct current stimulation (HD-tDCS), and cranial electrotherapy stimulation (CES) against a placebo/sham control condition in participants with PCC (19, 28, 43). Fatigue was the most consistently assessed domain. HD-tDCS yielded a significant between-group reduction in total fatigue scores with effects confined to cognitive and psychosocial subscales; physical fatigue showed no significant between-group difference (28). BFB produced greater reductions in general fatigue compared with sham, though total MFI improved in both groups(19). CES showed no between-group advantage on the Fatigue Assessment Scale; notably, the sham group improved significantly while the CES group did not (43). Anxiety showed heterogeneous between-group findings. HD-tDCS significantly reduced HAM-A scores vs sham, whereas neither BFB nor CES demonstrated significant between-group differences on their respective anxiety measures; both groups improved within-group in all 3 trials (19, 28, 43). Depression, sleep quality, and pain showed no significant between-group differences across the trials reporting these outcomes (19, 28, 43). Cognitive function was examined by only 1 trial, where BFB produced significantly greater improvement in immediate verbal recall and working memory maintenance vs sham (19).

*Oxygen-based interventions*. Four RCTs evaluated distinct oxygen-based modalities: intermittent hypoxia exposure (IHE) (31), hyperbaric oxygen therapy with 40 sessions (HBOT-40) (42), HBOT with 10 sessions (HBOT-10) (22), and a 4-arm dose-ranging normobaric/hyperbaric hyperoxia trial (NHHO) (38) against a placebo/sham (normoxia) control condition in participants with PCC. Functional exercise capacity showed heterogeneous between-group results. IHE produced significantly greater improvement in 6-minute walk distance (6MWD) than normoxia exposure (31). In contrast, HBOT-10 yielded no significant between-group difference in 6MWT and NHHO found no significant differences across all 4 oxygen dose arms (22, 38). Fatigue outcomes also varied. IHE produced significantly greater reductions in both the Fatigue Assessment Scale and Chalder Fatigue Scale vs normoxia (31). HBOT-40 demonstrated a significant between-group improvement in the SF-36 energy domain (42). HBOT-10 showed no significant between-group difference in any fatigue-related outcome (22). Spirometry was assessed by only 1 trial, where IHE produced significantly greater improvements in FVC, FEV_1_, FVC %pred, FEV_1_ %pred, and tidal volume compared with normoxia (31). Cognitive function findings were heterogeneous. HBOT-40 significantly improved global cognition, attention, and executive function, whereas NHHO found no significant between-group differences across 5 neurocognitive tests (38, 42). Health-related quality of life, assessed in 3 trials, consistently showed no significant between-group differences: RAND-36 physical functioning and role physical were null in HBOT-10, and VAS and EQ-5D-5L were null across all arms in NHHO; only HBOT-40 showed a significant between-group improvement in SF-36 physical limitations (31, 38, 42).

## DISCUSSION

This systematic review synthesized evidence from 27 randomized controlled trials to investigate the effectiveness of physical, psychological, and alternative interventions for patients with PCC. The findings indicate that multidisciplinary and exercise-based interventions are effective for promoting recovery in individuals with PCC. Cardiopulmonary rehabilitation and multimodal exercise interventions provide the strongest evidence for improving the primary outcomes of cardiorespiratory fitness and functional exercise capacity (6MWT distance) (21, 25–27, 29, 30, 32, 33, 35–37, 44–48). Consistent improvements were also observed across several secondary domains, most notably in fatigue severity, dyspnoea, and health-related quality of life (HRQoL). While psychological and cognitive interventions demonstrated efficacy in reducing fatigue and improving concentration, results for neuromodulation and oxygen-based therapies were more heterogeneous, suggesting that their clinical application may be dose-dependent or specific to certain patient phenotypes (19, 22, 28, 31, 38, 42, 43).

Our findings both corroborate and extend the expanding body of literature on non-pharmacological management for post-COVID-19 condition. The observed improvements in functional exercise capacity, measured via the 6-minute walk test, and the alleviation of dyspnoea align with previous meta-analyses demonstrating the robust efficacy of exercise-based cardiopulmonary rehabilitation (49–51). However, by restricting our eligibility criteria strictly to supervised, face-to-face interventions, this review resolves some of the conflicting evidence and “very low certainty” classifications reported in earlier reviews that pooled highly heterogeneous remote, hybrid, or unsupervised cohorts (50, 52)

Furthermore, this review extends the current literature by providing a more comprehensive, comparative analysis of alternative and psychological modalities. While standard exercise meta-analyses often report inconsistent or non-significant outcomes (52, 53) for fatigue our narrative synthesis highlights that specialized, symptom-titrated programmes and cognitive-behavioural therapy (CBT) offer clinically meaningful reductions in chronic fatigue. Conversely, our findings challenge the premature optimism surrounding biomedical and oxygen-based interventions. While earlier reviews suggested potential neurocognitive benefits from hyperbaric oxygen therapy (HBOT) (53), our integration of recent sham-controlled, multi-arm trials reveals no superior clinical benefit of oxygen therapy over placebo (22, 38). This suggests that previously reported improvements may be heavily influenced by strong placebo effects or natural symptom fluctuation over time.

The heterogeneity observed across trials may be interpreted through differences in intervention modality, supervision, and patient characteristics. Intensity also appears to be a moderating factor; while both moderate-intensity continuous training (MICT) and high-intensity interval training (HIIT) were effective, the latter was tolerated well only when combined with rigorous symptom monitoring to avoid triggering post-exertional malaise (PEM) (21, 29, 45). Participant characteristics, such as the severity of the initial infection, further contributed to differing results. Trials enrolling previously hospitalized patients often recorded more substantial absolute gains in functional capacity, potentially reflecting a lower baseline status and a greater degree of physiological deconditioning compared with non-hospitalized cohorts (27, 29, 35, 37). Additionally, the time elapsed since infection (ranging from 3 to 14 months) varied significantly, with some trials potentially capturing aspects of spontaneous recovery, particularly in control groups receiving self-management education (26, 29, 30, 36, 41, 48).

Methodological limitations of the included studies warrant careful consideration. Many trials were characterized by small sample sizes, which limits the generalizability of findings and the statistical power to detect small but clinically relevant differences in secondary outcomes (21, 22, 27, 33, 34, 36). The lack of blinding, inherent to most exercise and psychological interventions, introduces potential performance and detection bias (18, 25, 29, 30, 34, 39). Furthermore, the reliance on self-reported outcomes (PROMs) for complex constructs like “brain fog” and fatigue may not always correlate with objective physiological or neuropsychological markers (27, 32, 36, 41, 47). Long-term follow-up was infrequent, with most studies evaluating outcomes immediately post-intervention, thereby leaving the sustainability of benefits especially for fatigue and mental health largely unknown (18, 19, 39, 40, 44, 47). Finally, the recruitment of participants primarily through referral or social media may have resulted in a selection bias toward individuals with a higher motivation to exercise or those with more manageable symptom profiles, potentially excluding patients with severe fatigue (30, 34, 37, 48).

Two further constraints bear on the interpretation of effectiveness. Most trials reported multiple secondary outcomes without correction for multiple testing, so some of the statistically significant secondary findings reported here may represent false positives rather than true treatment effects. In addition, the substantial clinical and methodological heterogeneity across interventions precluded meta-analysis; this narrative synthesis can describe the direction and consistency of findings but cannot provide pooled effect estimates or a quantitative assessment of overall certainty. Taken together with the finding that only 5 of 27 trials (18.5%) were judged at low risk of bias, these limitations mean that the evidence base, while informative, does not currently support definitive conclusions concerning the effectiveness of any single intervention category.

The clinical relevance of these findings is profound, suggesting that a multidisciplinary and individualized approach is essential for addressing the multifaceted nature of PCC. For patients whose primary burden is respiratory (dyspnoea), integrated cardiopulmonary rehabilitation including inspiratory muscle training (IMT) and aerobic conditioning appears most beneficial (26, 30, 33, 35, 36, 44, 46). Conversely, for those with predominant fatigue and cognitive complaints, symptom-titrated exercise combined with cognitive-behavioural therapy (CBT) or structured lifestyle adjustments may prevent symptom exacerbation while improving functional status (32, 34, 39, 40, 54). Clinicians should prioritize personalized goal-setting and activity pacing, as standard “one-size-fits-all” recommendations were consistently less effective than supervised, tailored programsme (26, 30, 32, 45, 48). The inclusion of alternative modalities like balneotherapy or acupuncture may also serve as valuable adjuncts for managing musculoskeletal pain and somatic distress in specific subgroups. However, unlike oxygen-based interventions, the available studies evaluating alternative interventions did not include placebo-controlled comparisons, which should be considered when interpreting their reported benefits (18, 41).

When assessing the magnitude of observed effects, it is critical to distinguish between statistical significance and clinical relevance, and, in trials with an active comparator, between within-group change and a genuine between-group treatment effect. Several trials demonstrated between-group improvements that surpassed established minimal clinically important differences (MCIDs) relative to usual care or an inactive control. In the cardiorespiratory domain, cardiopulmonary rehabilitation produced a between-group 6MWT gain of 168 m against usual care, more than five-fold the 30-m MCID (35), and a sustained between-group gain of 39–47 m at 12 and 24 weeks in a further trial (30). By contrast, in trials comparing 2 active, non-blinded delivery formats of an otherwise similar rehabilitation programme, both arms improved by well above the MCID with no significant between-group difference, indicating comparable rather than superior efficacy of the experimental format (46). Reductions in dyspnoea (mMRC scale) against usual care likewise exceeded the 1-point MCID in several trials (18, 33, 35).

For fatigue, CBT produced a between-group CIS-fatigue reduction of 8.8 points (95% CI –11.9 to –5.8) compared with care as usual, a medium effect size (Cohen’s *d*=0.69) (34). However, several outcomes, particularly anxiety and global quality-of-life indices, showed statistically significant but modest between-group changes that often did not cross clinical thresholds, and in some active-comparator trials both arms crossed the relevant MCID with no significant between-group difference, suggesting that apparent gains partly reflect the passage of time, participation effects, or natural symptom fluctuation rather than a specific treatment effect (27, 41, 44)

Future research must prioritize larger, multi-centre randomized controlled trials with longer follow-up durations to determine the durability of rehabilitation effects. There is a critical need for studies employing objective outcome measures, such as neuroimaging (MRI), cardiopulmonary exercise testing (CPET) with gas exchange, and biomarkers of inflammation or mitochondrial function, to move beyond the limitations of self-report. Research should also focus on phenotyping approaches to identify which patients respond best to specific modalities, intensities, or delivery methods. Head-to-head comparisons of different rehabilitation durations and dosages, especially for neuromodulation and HBOT, are necessary to refine clinical protocols. Finally, the inclusion of more diverse populations and those with severe comorbidities will be vital for ensuring the external validity of future guidelines.

In conclusion, this narrative synthesis of 27 randomized controlled trials suggests that cardiopulmonary rehabilitation and multimodal exercise are promising interventions for improving cardiorespiratory fitness, functional capacity, and symptom burden in patients with PCC, with several trials showing between-group gains in 6-minute walk distance and dyspnoea perception that exceed established MCIDs when interventions are supervised and tailored to individual symptom tolerances. However, given the substantial clinical and methodological heterogeneity, small sample sizes, short follow-up durations, the low proportion of trials at low risk of bias (18.5%), and the absence of correction for multiple testing on secondary outcomes, this evidence does not support definitive conclusions regarding the effectiveness of any intervention category, and findings for neuromodulation and oxygen-based therapies remain particularly inconsistent. Adequately powered, low-risk-of-bias trials with longer follow-up and pre-specified primary outcomes are needed before these findings can inform clinical guidelines. In the interim, priorities for future research should include the development of phenotype-specific rehabilitation pathways, the integration of objective physiological markers to track recovery, and, where supervised rehabilitation is already offered as part of usual care, attention to whether gains are sustained and whether they exceed those achieved with standard care alone.

## Supplementary Material




