# Supplementary material for: EFFECTIVENESS OF PHYSICAL, PSYCHOLOGICAL, AND ALTERNATIVE INTERVENTIONS FOR POST-COVID-19 CONDITION: A SYSTEMATIC REVIEW
Source: J Rehabil Med. 2026 Oct 19;58:46279. doi: 10.2340/jrm.v58.46279 (PMC13685423; doi:10.2340/jrm.v58.46279)
Supplement: Supplementary file 1 [file JRM-58-46279-s1.pdf]

Table SI: Search Strategy

Date: 29.01.2026

Database	Search String	Results
Pubmed MEDLINE	("Post-Acute COVID-19 syndrome"[mh] OR "Post-COVID-19 syndrome"[tiab] OR "Post Covid"[tiab] OR "Post-COVID-19-Condition"[tiab] OR "long COVID"[tiab] OR "chronic COVID"[tiab] OR "post-acute sequelae of SARS-CoV-2 infection"[tiab] OR "Long Haul COVID-19"[tiab] OR "Long COVID"[tiab] OR "Post-COVID Conditions"[tiab] OR "Post-COVID Condition"[tiab])<br>**AND**<br>("Therapeutics"[mh] OR "Rehabilitation"[mh] OR "Physical Therapy Modalities"[mh] OR "Physical and Rehabilitation Medicine"[mh] OR "Rehabilitation Research"[mh] OR exercise[tiab] OR training[tiab] OR therapy[tiab] OR therapeutics[tiab] OR therap*[tiab] OR rehabilitation[tiab] OR "rehabilitation medicine"[tiab] OR "physical activity"[tiab] OR "physical medicine"[tiab] OR "physical therapy"[tiab] OR physiotherapy[tiab] OR "exercise training"[tiab] OR yoga[tiab] OR osteopathy[tiab] OR "transcranial stimulation"[tiab] OR "respiratory therapy"[tiab] OR "telehealth"[tiab] OR "aerobic training"[tiab] OR "resistance training"[tiab] OR "muscular resistance training"[tiab] OR HIT[tiab] OR HIIT[tiab] OR "high-intensity interval training"[tiab] OR "high-intensity aerobic training"[tiab] OR "home-exercise programme"[tiab] OR "multicomponent programme"[tiab] OR "multidisciplinary rehabilitation"[tiab] OR "physical reconditioning"[tiab] OR telerehabilitation[tiab] OR "breathing training"[tiab] OR "pulmonary rehabilitation"[tiab] OR "respiratory muscle training"[tiab] OR "inspiratory muscle training"[tiab] OR "expiratory muscle training"[tiab] OR "maximum inspiratory pressure"[tiab] OR "maximum expiratory pressure"[tiab] OR "respiratory rehabilitation"[tiab] OR "Complementary Therapies"[mh] OR "alternative medicine"[tiab] OR "complementary medicine"[tiab] OR "traditional medicine"[tiab] OR "integrative medicine"[tiab] OR naturopathy[tiab] OR "psychotherapy"[mh] OR "cognitive therapy"[tiab])<br>**AND**<br>("Comparative Effectiveness Research"[mh] OR "Treatment Outcome"[mh] OR "Outcome Assessment, Health Care"[mh] OR improvement[tiab] OR difference[tiab] OR efficacy[tiab] OR effectiveness[tiab] OR remedy[tiab] OR effect[tiab] OR development[tiab] OR "Quality Improvement"[mh] OR "Quality of life"[mh] OR "daily functioning"[tiab] OR "work capability"[tiab] OR "patient related outcome measures"[tiab] OR "patient-related outcome"[tiab] OR PROMs[tiab] OR PROs[tiab] OR "health-related quality"[tiab] OR "life quality"[tiab] OR "exercise capacity"[tiab] OR "functional exercise capacity"[tiab] OR "function test"[tiab] OR "event of treatment"[tiab] OR "Functional status"[mh] OR "disability evaluation"[mh] OR "outcome assessment"[mh] OR "patient-reported outcome measure"[mh] OR "long-term symptoms"[tiab] OR "respiratory function"[tiab])	n = 3.626

Web of Science	TS=("Post-Acute COVID-19 syndrome" OR "Post-COVID-19 syndrome" OR "Post Covid" OR "Post-COVID-19-Condition" OR "long COVID" OR "chronic COVID" OR "post-acute sequelae of SARS-CoV-2 infection" OR "Long Haul COVID-19" OR "Long COVID" OR "Post-COVID Conditions" OR "Post-COVID Condition")<br>**AND**<br>TS=("Therapeutics" OR "Rehabilitation" OR "Physical Therapy Modalities" OR "Physical and Rehabilitation Medicine" OR "Rehabilitation Research" OR exercise OR training OR therapy OR therapeutics OR therap* OR rehabilitation OR "rehabilitation medicine" OR "physical activity" OR "physical medicine" OR physical therapy OR physiotherapy OR "exercise training" OR yoga OR osteopathy OR "transcranial stimulation" OR "respiratory therapy" OR "telehealth" OR "aerobic training" OR "resistance training" OR "muscular resistance training" OR HIT OR HIIT OR "high-intensity interval training" OR "high-intensity aerobic training" OR "home-exercise programme" OR "multicomponent programme" OR "multidisciplinary rehabilitation" OR "physical reconditioning" OR telerehabilitation OR "breathing training" OR "Pulmonary rehabilitation" OR "Respiratory muscle training" OR "Inspiratory muscle training" OR "Expiratory muscle training" OR "Maximum inspiratory pressure" OR "Maximum expiratory pressure" OR "Respiratory rehabilitation" OR "Complementary Therapies" OR "alternative medicine" OR "complementary medicine" OR "traditional medicine" OR "integrative medicine" OR naturopathy OR psychotherapy OR "cognitive therapy")<br>**AND**<br>TS=("Comparative Effectiveness Research" OR "Treatment Outcome" OR "Outcome Assessment, Health Care" OR improvement OR difference OR efficacy OR effectiveness OR remedy OR effect OR development OR "Quality Improvement" OR "Quality of life" OR "daily functioning" OR "work capability" OR "patient related outcome measures" OR "patient-related outcome" OR PROMs OR PROs OR "health-related quality" OR "life quality" OR "exercise capacity" OR "functional exercise capacity" OR "function test" OR "event of treatment" OR "Functional status" OR "disability evaluation" OR "outcome assessment" OR "patient-reported outcome measure" OR "long-term symptoms" OR "respiratory function")	n = 3.223
Scopus	TITLE-ABS("Post-Acute COVID-19 syndrome" OR "Post-COVID-19 syndrome" OR "Post Covid" OR Post-COVID-19-Condition OR "long COVID" OR "chronic COVID" OR "post-acute sequelae of SARS-CoV-2 infection" OR "Long Haul COVID-19" OR "Long COVID" OR "Post-COVID Conditions" OR "Post-COVID Condition")<br>**AND**<br>TITLE-ABS("Therapeutics" OR "Rehabilitation" OR "Physical Therapy Modalities" OR "Physical and Rehabilitation Medicine" OR "Rehabilitation Research" OR exercise OR training OR therapy OR therapeutics OR therap* OR rehabilitation OR "rehabilitation medicine" OR "physical activity" OR "physical medicine" OR physical therapy OR physiotherapy OR "exercise training" OR yoga OR osteopathy OR "transcranial stimulation" OR "respiratory	n = 1.853

	therapy" OR "telehealth" OR "aerobic training" OR "resistance training" OR "muscular resistance training" OR HIT OR HIIT OR "high-intensity interval training" OR "high-intensity aerobic training" OR "home-exercise programme" OR "multicomponent programme" OR "multidisciplinary rehabilitation" OR "physical reconditioning" OR telerehabilitation OR "breathing training" OR "Pulmonary rehabilitation" OR "Respiratory muscle training" OR "Inspiratory muscle training" OR "Expiratory muscle training" OR "Maximum inspiratory pressure" OR "Maximum expiratory pressure" OR "Respiratory rehabilitation" OR "Complementary Therapies" OR "alternative medicine" OR "complementary medicine" OR "traditional medicine" OR "integrative medicine" OR naturopathy OR psychotherapy OR "cognitive therapy")<br>**AND**<br>TITLE-ABS("Comparative Effectiveness Research" OR "Treatment Outcome" OR "Outcome Assessment, Health Care" OR improvement OR difference OR efficacy OR effectiveness OR remedy OR effect OR development OR "Quality Improvement" OR "Quality of life" OR "daily functioning" OR "work capability" OR "patient related outcome measures" OR "patient-related outcome" OR PROMs OR PROs OR "health-related quality" OR "life quality" OR "exercise capacity" OR "functional exercise capacity" OR "function test" OR "event of treatment" OR "Functional status" OR "disability evaluation" OR "outcome assessment" OR "patient-reported outcome measure" OR "long-term symptoms" OR "respiratory function")	
PsycINFO via APA	AB ("Post-Acute COVID-19 syndrome" OR "Post-COVID-19 syndrome" OR "Post Covid" OR "Post-COVID-19-Condition" OR "long COVID" OR "chronic COVID" OR "post-acute sequelae of SARS-CoV-2 infection" OR "Long Haul COVID-19" OR "Post-COVID Conditions" OR "Post-COVID Condition")<br>**AND**<br>AB ("Therapeutics" OR "Rehabilitation" OR "Physical Therapy Modalities" OR "Physical and Rehabilitation Medicine" OR "Rehabilitation Research" OR exercise OR training OR therapy OR therapeutics OR therap* OR rehabilitation OR "rehabilitation medicine" OR "physical activity" OR "physical medicine" OR physical therapy OR physiotherapy OR "exercise training" OR yoga OR osteopathy OR "transcranial stimulation" OR "respiratory therapy" OR "telehealth" OR "aerobic training" OR "resistance training" OR "muscular resistance training" OR HIT OR HIIT OR "high-intensity interval training" OR "high-intensity aerobic training" OR "home-exercise programme" OR "multicomponent programme" OR "multidisciplinary rehabilitation" OR "physical reconditioning" OR telerehabilitation OR "breathing training" OR "Pulmonary rehabilitation" OR "Respiratory muscle training" OR "Inspiratory muscle training" OR "Expiratory muscle training" OR "Maximum inspiratory pressure" OR "Maximum expiratory pressure" OR "Respiratory rehabilitation" OR "Complementary Therapies" OR "alternative medicine" OR "complementary medicine" OR "traditional medicine" OR "integrative medicine" OR naturopathy OR psychotherapy OR "cognitive behavioral therapy" OR “counseling”)	n = 191

	**AND**<br>AB ("Comparative Effectiveness Research" OR "Treatment Outcome" OR "Outcome Assessment, Health Care" OR improvement OR difference OR efficacy OR effectiveness OR remedy OR effect OR development OR "Quality Improvement" OR "Quality of life" OR "daily functioning" OR "work capability" OR "patient related outcome measures" OR "patient-related outcome" OR PROMs OR PROs OR "health-related quality" OR "life quality" OR "function test" OR "event of treatment" OR "Functional status" OR "disability evaluation" OR "outcome assessment" OR "patient-reported outcome measure" OR "long-term symptoms" OR "respiratory function")	

Table SII: List of excluded full-text articles

Author and year	Title	Reason for exclusion
Abo Elyazed et al., 2024	Diaphragmatic strengthening exercises for patients with post COVID-19 condition after mild-to-moderate acute COVID-19 infection: A randomized controlled study	wrong setting
Aggarwal and Chanda, 2025	Effects of thoracic spinal manipulation in long COVID patients: A randomised controlled trial.	wrong outcome
Berry et al., 2025	Resistance Exercise Therapy After COVID-19 Infection: A Randomized Clinical Trial	wrong population
Calvache-Mateo et al., 2025	Effectiveness of a Pain Informed Movement Program in Individuals With Post-COVID-19 Condition: A Randomized Controlled Trial	wrong setting
Campos et al., 2024	Evaluation of vascular photobiomodulation for orofacial pain and tension type headache following COVID 19 in a pragmatic randomized clinical trial	wrong population
Cunha et al., 2025	Whole body vibration exercise effects on exercise capacity and muscle strength in long Covid-19 patients: A randomized clinical trial	wrong outcome
Damiano et al., 2024	Learning from the COVID-19 challenge: an Italian RCT study on an intervention's effectiveness in reducing mental health symptoms in hospitalized patients	wrong population
Daynes et al., 2025	Post-Hospitalisation COVID-19 Rehabilitation (PHOSP-R): a randomised controlled trial of exercise-based rehabilitation	wrong setting
Del Corral et al., 2025	Effect of respiratory rehabilitation on quality of life in individuals with post-COVID-19 symptoms: A randomised controlled trial	wrong setting
Hausswirth et al., 2023	Positive Impacts of a Four-Week Neuro-Meditation Program on Cognitive Function in Post-Acute Sequelae of COVID-19 Patients: A Randomized Controlled Trial	wrong population

Ibrahim, Dewir et al., 2023	Influences of high vs. low-intensity exercises on muscle strength, function, and quality of life in post-COVID-19 patients with sarcopenia: a randomized controlled trial	wrong population
Ibrahim, Hussein et al., 2023	A randomized controlled trial examining the impact of low vs. moderate-intensity aerobic training in post-discharge COVID-19 older subjects	wrong population
Karelova et al., 2025	Personalized Approach in Complex Rehabilitation of Patients after COVID-19 Pneumonia Using Hypoxia-Hyperoxia Therapy: a Randomized Study	Foreign language
Klírová et al., 2024	Transcranial direct current stimulation tDCS in the treatment of neuropsychiatric symptoms of long COVID	wrong population
Liu et al., 2025	Effects of inspiratory muscle training on respiratory function, psychology and sleep in post- COVID- 19 patients: A randomized controlled trial	Foreign language
Mabrouk et al., 2025	The impact of aerobic and balance exercises on anxiety and dizziness in post-COVID-19 patients: a randomised clinical trial	wrong population
Murano et al., 2025	The impact of Mindfulness-based stress reduction on Covid-19 survivors. A randomized controlled trial	wrong population
Nagy et al., 2022	Influence of Manual Diaphragm Release Technique Combined with Inspiratory Muscle Training on Selected Persistent Symptoms in Men with Post-Covid-19 Syndrome: A Randomized Controlled Trial	wrong population
Nambi et al., 2022	Comparative effectiveness study of low versus high-intensity aerobic training with resistance training in community-dwelling older men with post-COVID 19 sarcopenia: A randomized controlled trial	wrong population
Nerli et al., 2024	Brief Outpatient Rehabilitation Program for Post-COVID-19 Condition: A Randomized Clinical Trial	wrong population

P et al., 2025	Effects of Bhramari and Sheetali Pranayama on Cardio Respiratory Function in Post-COVID Patients: A Randomised Controlled Study	wrong outcome
Paithoon et al., 2025	Effectiveness of Herbal Steam Bath Therapy for Quality of Life and Sleep Quality in Post-Covid-19 Patients	wrong population
Rasmussen et al., 2024	Impact of a 12-week high-intensity interval training intervention on cardiac structure and function after COVID-19 at 12-month follow-up	wrong population
Romanet et al., 2023	Effectiveness of exercise training on the dyspnoea of individuals with long COVID: A randomised controlled multicentre trial	wrong population
Sánchez Milá et al., 2024	Effects of a respiratory and neurological rehabilitation treatment plan in post Covid-19 affected university students: Randomized clinical study	wrong setting
Sun et al., 2026	The effect of Tai Chi exercise on physical and psychological health of college students with persistent fatigue after COVID-19 infection: A randomized controlled trial	wrong population
Vidal et al., 2025	Transcranial direct current stimulation plus cognitive training for cognitive symptoms in patients with post-acute sequelae of SARS-CoV-2 infection: A randomized, double-blind, sham-controlled trial	wrong population
Zissler et al., 2025	Acute Effects of Osteopathic Treatment in Long COVID-19 Patients with Fatigue Symptoms: A Randomized, Controlled Trial	wrong study design

Reference List

1. Abo Elyazed, T. I., Abd El-Moneim Abd El-Hakim, A., Saleh, O. I., Sonbol, M. M. F., Eid, H. A., Moazen, E. M., Alhassoon, M. H., and Elfeky, S. E. F. (2024). Diaphragmatic strengthening exercises for patients with post COVID-19 condition after mild-to-moderate acute COVID-19 infection: A randomized controlled study. J. Rehabil. Med. 56, jrm25491. DOI: 10.2340/jrm.v56.25491.

2. Aggarwal, A., and Chanda, A. (2025). Effects of thoracic spinal manipulation in long COVID patients: A randomised controlled trial. Respir. Med. *250,* 108503. DOI: 10.1016/j.rmed.2025.108503.
3. Berry, C., McKinley, G., Bayes, H.K., Anderson, D., Lang, C.C., Gill, A., Morrow, A., Sykes, R., Taggart, D., Kamdar, A., Welsh, P., Dawkes, S., McConnachie, A., and Gray, S.R. (2025). Resistance Exercise Therapy After COVID-19 Infection: A Randomized Clinical Trial. JAMA Network Open DOI: 10.1001/jamanetworkopen.2025.34304.
4. Calvache-Mateo, A., Navas-Otero, A., Raya-Benítez, J., Martín-Núñez, J., Heredia-Ciuró, A., Ortiz-Rubio, A., & Valenza, M. C. (2025). Effectiveness of a Pain Informed Movement Program in Individuals With Post-COVID-19 Condition: A Randomized Controlled Trial. Archives of physical medicine and rehabilitation, 106(11), 1636–1644. https://doi.org/10.1016/j.apmr.2025.05.008
5. Campos, M.C.V., Schuler, S.S.V., Lacerda, A.J., Mazzoni, A.C., Silva, T., Rosa, F.C.S., Martins, M.D., Fernandes, K.P.S., Fonseca, E.S., Mesquita-Ferrari, R.A., ACRT, H., Bussadori, S.K., and Motta, L.J. (2024). Evaluation of vascular photobiomodulation for orofacial pain and tension type headache following COVID 19 in a pragmatic randomized clinical trial. Scientific Reports *14,* 31138. DOI: 10.1038/s41598-024-82412-9.
6. Cunha, B.L.M., Policarpo, J.H., Silva, J.R.D., Porfírio, P.V., LRDS, F., Leitão, C.C.S., and Marinho, P.E.M. (2025). Whole body vibration exercise effects on exercise capacity and muscle strength in long Covid-19 patients: A randomized clinical trial. J. Bodywork Movement Ther. *44,* 494–500. DOI: 10.1016/j.jbmt.2025.06.013.
7. Damiano, R., Giulia, G., Antonietta, B., Annalisa, D., Anna, R., Francesca, B., Matteo, M., Giacomel, I., Stefano, P., Musella, V., Lavinia, B., and Catherine, K. (2024). Learning from the COVID-19 challenge: an Italian RCT study on an intervention's effectiveness in reducing mental health symptoms in hospitalized patients. Current Psychology *43,* 35772–35784. DOI: 10.1007/s12144-024-06907-4.
8. Daynes, E., Evans, R. A., Greening, N. J., Bishop, N. C., Yates, T., Lozano-Rojas, D., Ntotsis, K., Richardson, M., Baldwin, M. M., Hamrouni, M., Hume, E., McAuley, H., Mills, G., Megaritis, D., Roberts, M., Bolton, C. E., Chalmers, J. D., Chalder, T., Docherty, A. B., Elneima, O., … PHOSP-COVID Study Collaborative Group (2025). Post-Hospitalisation COVID-19 Rehabilitation (PHOSP-R): a randomised controlled trial of exercise-based rehabilitation. The European respiratory journal, 65(5), 2402152. https://doi.org/10.1183/13993003.02152-2024
9. Del Corral, T., Fabero-Garrido, R., Plaza-Manzano, G., Izquierdo-García, J., López-Sáez, M., García-García, R., and López-de-Uralde-Villanueva, I. (2025). Effect of respiratory rehabilitation on quality of life in individuals with post-COVID-19 symptoms: A randomised controlled trial. Annals of Physical and Rehabilitation Medicine *68,* 101920. DOI: 10.1016/j.rehab.2024.101920.

10. Hausswirth, C., Schmit, C., Rougier, Y., and Coste, A. (2023). Positive Impacts of a Four-Week Neuro-Meditation Program on Cognitive Function in Post-Acute Sequelae of COVID-19 Patients: A Randomized Controlled Trial. International Journal of Environmental Research and Public Health *20,* DOI: 10.3390/ijerph20021361.
11. Ibrahim, A.A., Dewir, I.M., ST, A.E.K., Ragab, M.M., Abdel-Fattah, M.S., and Hussein, H.M. (2023). Influences of high vs. low-intensity exercises on muscle strength, function, and quality of life in post-COVID-19 patients with sarcopenia: a randomized controlled trial. Eur. Rev. Med. Pharmacol. Sci. *27,* 9530–9539. DOI: 10.26355/eurrev_202310_34126.
12. Ibrahim, A.A., Hussein, H.M., Ali, M.S., Kanwal, R., Acar, T., Shaik, D.H., Alghamdi, W., and Althomali, O.W. (2023). A randomized controlled trial examining the impact of low vs. moderate-intensity aerobic training in post-discharge COVID-19 older subjects. Eur. Rev. Med. Pharmacol. Sci. *27,* 4280–4291. DOI: 10.26355/eurrev_202305_32338.
13. Karelova, N.Y., Kulishova, T.V., Kharchenko, S.S., and Babushkin, I.E. (2025). Personalized Approach in Complex Rehabilitation of Patients after COVID-19 Pneumonia Using Hypoxia-Hyperoxia Therapy: a Randomized Study. Vestnik Vosstanovitel'noj Mediciny *24,* 34–46. DOI: 10.38025/2078-1962-2025-24-5-34-46.
14. Klírová, M., Adamová, A., Biačková, N., Laskov, O., Renková, V., Stuchlíková, Z., Odnohová, K., and Novák, T. (2024). Transcranial direct current stimulation (tDCS) in the treatment of neuropsychiatric symptoms of long COVID. Scientific Reports *14,* 2193. DOI: 10.1038/s41598-024-52763-4.
15. Liu, X., Li, X., and Jiang, H. (2025). Effects of inspiratory muscle training on respiratory function， psychology and sleep in post- COVID-19 patients： A randomized controlled trial. Chinese Journal of Rehabilitation Medicine *40,* 900–908. DOI: 10.3969/j.issn.1001-1242.2025.06.015.
16. Mabrouk, M.I., Abdalbary, S.A., Abdelmonem, A.F., Abdelhakiem, N.M., Ahmed, A.A., Gelany, F.M., and Abd El Rahim, A.A. (2025). The impact of aerobic and balance exercises on anxiety and dizziness in post-COVID-19 patients: a randomised clinical trial. Physiotherapy Quarterly *33,* 71–79. DOI: 10.5114/pq/190527.
17. Murano, L., Damico, V., Cossalter, L., Riggio, M., Calabresi, F., Zappia, L., and Margosio, V. (2025). The impact of Mindfulness-based stress reduction on Covid-19 survivors. A randomized controlled trial. Ann. Ig. *37,* 350–364. DOI: 10.7416/ai.2025.2678.
18. Nagy, E.N., Elimy, D.A., Ali, A.Y., Ezzelregal, H.G., and Elsayed, M.M. (2022). INFLUENCE OF MANUAL DIAPHRAGM RELEASE TECHNIQUE COMBINED WITH INSPIRATORY MUSCLE TRAINING ON SELECTED PERSISTENT SYMPTOMS IN MEN WITH POST-COVID-19 SYNDROME: A RANDOMIZED CONTROLLED TRIAL. J. Rehabil. Med. *54,* DOI: 10.2340/jrm.v54.3972.
19. Nambi, G., Abdelbasset, W.K., Alrawaili, S.M., Elsayed, S.H., Verma, A., Vellaiyan, A., Eid, M.M., Aldhafian, O.R., Nwihadh, N.B., and Saleh, A.K. (2022). Comparative effectiveness study of low versus high-intensity aerobic training with resistance training in community-dwelling older men with post-COVID 19 sarcopenia: A randomized controlled trial. Clin. Rehabil. *36,* 59–68. DOI: 10.1177/02692155211036956.

20. Nerli, T.F., Selvakumar, J., Cvejic, E., Heier, I., Pedersen, M., Johnsen, T.L., and Wyller, V.B.B. (2024). Brief Outpatient Rehabilitation Program for Post-COVID-19 Condition: A Randomized Clinical Trial. JAMA Network Open *7,* e2450744. DOI: 10.1001/jamanetworkopen.2024.50744.
21. P, R., Saravanan, A., Maheshkumar, K., ThamaraiSelvi, K., Praba, P.K., and Prabhu, V. (2025). Effects of Bhramari and Sheetali Pranayama on Cardio Respiratory Function in Post-COVID Patients: A Randomised Controlled Study. Annals of Neurosciences 09727531241306849. DOI: 10.1177/09727531241306849.
22. Paithoon, R., Sriyakul, K., Tungsukruthai, P., Srikaew, N., Sawangwong, P., Tungsukruthai, S., and Phetkate, P. (2025). Effectiveness of Herbal Steam Bath Therapy for Quality of Life and Sleep Quality in Post-Covid-19 Patients. Natural and Life Sciences Communications *24,* DOI: 10.12982/NLSC.2025.009.
23. Rasmussen, I.E., Løk, M., Durrer, C.G., Lytzen, A.A., Foged, F., Schelde, V.G., Budde, J.B., Rasmussen, R.S., Høvighoff, E.F., Rasmussen, V., Lyngbæk, M., Jønck, S., Krogh-Madsen, R., Lindegaard, B., Jørgensen, P.G., Køber, L., Vejlstrup, N., Pedersen, B.K., Ried-Larsen, M., Lund, M.A.V., Berg, R.M.G., and Christensen, R.H. (2024). Impact of a 12-week high-intensity interval training intervention on cardiac structure and function after COVID-19 at 12-month follow-up. Exp. Physiol. DOI: 10.1113/EP092099.
24. Romanet, C., Wormser, J., Fels, A., Lucas, P., Prudat, C., Sacco, E., Bruel, C., Plantefève, G., Pene, F., Chatellier, G., and Philippart, F. (2023). Effectiveness of exercise training on the dyspnoea of individuals with long COVID: A randomised controlled multicentre trial. Annals of Physical and Rehabilitation Medicine *66,* 101765. DOI: 10.1016/j.rehab.2023.101765.
25. Sánchez Milá, Z., Rodríguez Sanz, D., Martín Nieto, A., Jiménez Lobo, A., Ramos Hernández, M., Campón Chekroun, A., Frutos Llanes, R., Barragán Casas, J. M., and Velázquez Saornil, J. (2024). Effects of a respiratory and neurological rehabilitation treatment plan in post COVID-19 affected university students: Randomized clinical study. Chronic Respir. Dis. 21, 1–10. DOI: 10.1177/14799731241255967
26. Sun, J., Yao, K., Chu, H., Chen, J., Du, C., Li, B., Yu, Z., Zhang, Z., and Chen, T. (2026). The effect of Tai Chi exercise on physical and psychological health of college students with persistent fatigue after COVID-19 infection: A randomized controlled trial. Public Health *251,* 106105. DOI: 10.1016/j.puhe.2025.106105.
27. Vidal, K.S., Cavendish, B.A., Goerigk, S., Pita Batista, M., Oliveira Lima, A.R., Pinto, B.S., Neto Domingos, A.A., de Sousa, J.P., Pelosof, R., Bertola, L., Silva, V., Suemoto, C.K., Razza, L.B., Bikson, M., Pilloni, G., Charvet, L., Silva, P.H.R., and Brunoni, A.R. (2025). Transcranial direct current stimulation plus cognitive training for cognitive symptoms in patients with post-acute sequelae of SARS-CoV-2 infection: A randomized, double-blind, sham-controlled trial. Brain Stimulation *18,* 1608–1616. DOI: 10.1016/j.brs.2025.08.018.
28. Zissler, U.M., Poehlmann, T., Gloeckl, R., Ibrahim, S., Klupsch, K., Schneeberger, T., Jarosch, I., and Koczulla, A.R. (2025). Acute Effects of Osteopathic Treatment in Long COVID-19 Patients with Fatigue Symptoms: A Randomized, Controlled Trial. Journal of Clinical Medicine *14,* DOI: 10.3390/jcm14176066.
